# Addressing schoolteacher food and nutrition-related health and wellbeing: a scoping review of the food and nutrition constructs used across current research

**DOI:** 10.1186/s12966-023-01502-5

**Published:** 2023-09-12

**Authors:** Tammie Jakstas, Berit Follong, Tamara Bucher, Andrew Miller, Vanessa A. Shrewsbury, Clare E. Collins

**Affiliations:** 1 School of Health Sciences, College of Health, Medicine and Wellbeing, The University of Newcastle, Callaghan, NSW 2308 Australia; 2 Food and Nutrition Research Program, Hunter Medical Research Institute, New Lambton Heights, NSW 2305 Australia; 3 National Institute for Health Innovation, The University of Auckland, Auckland, 1010 New Zealand; 4 School of Environmental and Life Sciences, College of Engineering, Science and Environment, The University of Newcastle, Callaghan, NSW 2308 Australia; 5 School of Education, College of Human and Social Futures, The University of Newcastle, Callaghan, NSW 2308 Australia; 6 Priority Research Centre for Teachers and Teaching, The University of Newcastle, Callaghan, NSW 2308 Australia

**Keywords:** Teachers, Scoping review, Wellbeing, Food, Nutrition, Diet, Eating, Mental health

## Abstract

**Background:**

Teachers form a large and essential workforce globally. Their wellbeing impacts personal health-related outcomes with flow on effects for the health, and wellbeing of their students. However, food and nutrition (FN) interventions that include teachers, typically neglect the impact of personal FN factors on a teachers’ ability to achieve optimal nutrition-related health and wellbeing, and successfully fulfil their professional FN roles as health promoters, gate keepers, educators’, and role models. The aim of this review was to scope FN constructs that have been studied internationally regarding teacher FN-related health and wellbeing.

**Methods:**

Six databases were searched, and papers extracted in June/July 2021. Eligibility criteria guided by the population, concept, context mnemonic included studies published after 2000, in English language, with an aspect of personal FN-related health and wellbeing, among in-service (practising) and pre-service (training), primary, and secondary teachers. Screening studies for inclusion was completed by two independent researchers with data extraction piloted with the same reviewers and completed by lead author, along with complete descriptive and thematic analysis.

**Results:**

Ten thousand six hundred seventy-seven unique articles were identified with 368 eligible for full text review and 105 included in final extraction and analysis. Sixty-nine descriptive studies were included, followed by 35 intervention studies, with the main data collection method used to assess both personal and professional FN constructs being questionnaires (*n* = 99 papers), with nutrition knowledge and dietary assessment among the most commonly assessed.

**Conclusion:**

FN constructs are used within interventions and studies that include teachers, with diversity in constructs included and how these terms are defined. The evidence from this scoping review can be used to inform data collection and evaluation in future epidemiological and interventional research that addresses teacher FN-related health and wellbeing.

**Supplementary Information:**

The online version contains supplementary material available at 10.1186/s12966-023-01502-5.

## Background

Teachers’ health and wellbeing, including food and nutrition (FN) practices are influenced by professional workload and school environment [1], while they in turn have an influence on the students within their care as role models [2], health promoters [3, 4], gate keepers [5], and FN educators [6]. While high rates of teacher work-related stress and burnout have been identified as contributors to teacher turnover, highlighting the impact of teacher wellbeing on performance of work-related tasks, the influence of FN practices on teacher wellbeing has not been explored [1, 7–9].

Indicators and predictors of food choices and eating behaviours are unique to everyone and acknowledged as complex in the Determinates of Nutrition and Eating (DONE) Framework, which identifies 51 determinate groups across four key domains of individual, interpersonal, environmental and policy [10, 11]. FN constructs known to be indicators of healthy dietary patterns such as positive food agency, cooking skills and food skills [12, 13] are increasingly used along with forms of food literacy measures [14], in mental health and wellbeing interventions, targeting an individuals’ wellbeing and dietary outcomes [15, 16]. Despite this, few studies with teacher participants include an examination of FN constructs beyond measuring dietary assessment and/or nutrition knowledge which provides limited information on the overall influence of FN to the related health and wellbeing of teachers.

Poor diet quality, specifically low intake of vegetables, fruit and wholegrains are well established as risk factors of chronic disease and contributors to global burden of disease [17]. To this, FN-related constructs such as cooking confidence and diet quality are increasingly included in research that considers the links between mental health outcomes, including depression and anxiety [18, 19]. With teacher wellbeing known to be impacted by stress and burnout, and the growing evidence supporting the role of diet and potential benefits of culinary practices in mediating mental health outcomes, there is a need to consider a greater focus be given to a teachers’ personal FN-related health and wellbeing.

Teachers need support and education on how to optimise their own FN-related health and wellbeing to help them in fulfilling their professional FN roles as healthy role models and advocates for the students in their care [20, 21]. Previous review studies have explored the impact of work-related factors on the health and wellbeing of early learning educators; but not schoolteachers, with limited review of FN beyond brief dietary indicators [22–26]. Although, more recent reviews and research have looked at the concept of, and/or contributors of wellbeing in primary and secondary schoolteachers [1, 7, 9, 27–32], including mental health, stress, and burnout, they have not considered the influence or role of FN factors. One systematic review and meta-analysis on teacher nutrition education professional development interventions was identified, but it did not consider the impact of this education on teacher wellbeing [33]. This highlights a clear gap to investigate research that has included aspects of teacher FN, how the FN constructs were measured and what, if anything they can tell us about the potential influence FN factors have on teacher-related health and wellbeing.

Therefore, the current scoping review aims to summarise the range of FN constructs included across research on teacher’s personal FN-related health and wellbeing. The review will map evidence on teacher FN-related health and wellbeing and how this has been evaluated to inform future research.

## Methods

A scoping review methodology was selected to both enable research from a diverse collection of areas across education, and health, while providing a structed yet iterative process that provided a clear review framework with the flexibility to refine parameters as references were collected and information sourced. The term FN-related health and wellbeing is used within the current review to encompass the complexity of individual, interpersonal, environmental and policy related constructs that influence an individuals’ FN decisions and healthy eating behaviours. For the purposes of this review a distinction is made between personal and professional FN constructs, as outlined in Table 1. Personal FN constructs relate to the individual teachers, primarily in their personal lives, even though these may have downstream effects on student FN factors e.g., through role modelling eating behaviours or a capability to transfer deep FN-related knowledge and skills. Professional FN constructs are defined here as those specifically related to the teachers’ professional role, even though these may potentially also influence personal FN.

**Table 1:** Personal and professional FN constructs defined

**Personal FN constructs**	**Professional FN constructs**
• Measure of dietary intake [34, 35] [Includes: Food Frequency Questionnaires, Dietary screeners that capture an aspect of diet quality (i.e., fats only or fruit and vegetable intake)] • Food habits [36] • Eating behaviours at school [37] • Food and cooking skills and confidence [38] • Food literacy [39] • Nutrition knowledge [40] (Specific to teacher personal needs) • Nutrition self-efficacy [41] • Food agency [13] • Food attitudes [42] • Behavioral intentions connected to nutrition and healthy eating [42]	• Classroom food practices [43] (May include use of food as a reward, role modeling of food and nutrition practices) • Nutrition teaching self-efficacy [44] • Health promoter [3] • Gatekeeper [5] • Policy implementation [45] • Nutrition knowledge [46] (Specific to student health and nutrition needs or student education provision).

### Design

A scoping review protocol was developed, guided by the Joanna Briggs Institute (JBI) guidelines [47], the preferred reporting items for systematic reviews and meta-analysis extension for scoping reviews (PRISMA-Scr) checklist [48, 49] (Additional file 1) along with complementary papers [50, 51] and research guidelines [52].

### Eligibility criteria

Early learning preservice and in-service teacher/educators were not included within this review as earlier scoping reviews exploring this population were identified [22–26] and for the differences noted between school-based teachers and early learning teacher/educators. Many studies identified in screening often used the terms educators and teachers interchangeably making it difficult to distinguish between them, with notable differences recognised in in their daily responsibilities or workload and the training required to become an early childhood teacher (e.g., a 3–4-year university degree) or an early childhood educator (e.g., a variety of Technical and Further Education (TAFE) certificates or diplomas). Table 2 provides a full summary of the inclusion and exclusion criteria.

**Table 2:** Scoping review inclusion and exclusion criteria

	**Inclusion criteria**	**Exclusion criteria**
**Sources**		
Types	Published peer reviewed journal articles.	Study protocols, conference abstracts, posters and published dissertations, editorials, news bulletins, policy documents or policy briefs.
Date of publication	Published after 2000	Published before 2000
Language	English	Articles not written in English
Subjects	Studies in humans	Studies in animals
**Participants**	Preservice and in-service primary and secondary (including relevant international equivalents) schoolteachers of all ages, sociodemographic status and teaching area or learning disciplines. Interventions conducted in adolescents, children, students or whole school interventions with included teacher training or intervention will also be included, yet they must clearly report on the teacher component or outcomes. This includes studies that focus on “school staff” but clearly state the inclusion of teachers within the participant sample. Combined data that includes teacher participants will be included without the extraction of outcome results in instances where teacher participant specific data is not provided separately.	Preservice and in-service teachers in early learning, childcare, head start, nursery, or kindergartens. Tertiary teachers including technical colleges, after school program educators and teachers, volunteer teachers in community-based school programs. School based dietitians, FN professionals working in schools.
**Concept**	Studies or research that report on constructs of either/and/or: • Teacher FN wellbeing • FN training provision AND have at least one personal FN factor included. An overview of personal and professional FN factors is included in Table 1.	Studies or research that report on constructs of: 1. Only teacher professional FN (e.g., nutrition education self-efficacy or those that focused only on teacher nutrition knowledge relevant to student health, without reference to any personal FN factors). 2. Teacher wellbeing programs or training without FN relevant components. 3. Student or whole school wellbeing interventions without teacher training or outcomes collected.
**Context**	• Schools, universities, colleges, online formats • Training programs through outside or community bodies that deliver relevant teacher training or health intervention • Teacher FN training within a student intervention or school environment study will also be considered.	

### Literature search strategy

In June/July 2021 six databases were searched: PsychInfo, ERIC via PROQUEST, CINAHL, Medline, Embase, Scopus. Database specific search strategies were developed in consultation with two senior University of Newcastle librarians’ using the population, concept, context, (PCC) mnemonic [47]. The Medline search strategy is shown in Table 3, with all database search strategies documented in Additional file 2. The reference list of included papers was screened for additional eligible papers.

**Table 3:** Medline search strategy

**PCC Element**	**Search terms**	**Field**
Context	Schools	Key word/title/abstract
Context	(primary or elementary or headstart or early childhood or secondary or high or middle or school) near3 (teacher* or schoolteacher* or educator*)	Key word/title/abstract
Population	(early career or inservice or in-service or pre-service or preservice or prospective or student) near3 teacher*)	Key word/title/abstract
Concept	((food* or nutri* or diet* or cook* or eat*) near3 (belief* or attitude* or habit* or quality* or literac* or health or educat* or program or train* or wellbeing or well being or culinary or curricul* or knowledge or status or polic* or skill* or agency or pedagogy or behavio?r* or practic* or experience* or motivat* or self efficacy or self perception or classroom* or environment or model* or advocat*))	Key word/title/abstract
5	1 or 2 or 3	
6	4 and 5	
7	limit 6 to english	

### Study selection

Screening of papers was conducted by two independent reviewers (BF, TJ) using Covidence systematic review software, Veritas Health Innovation, Melbourne, Australia, available at www.covidence.org. Title and abstract screening, and full text screening were conducted by BF, TJ with conflicts resolved by discussion and by a third independent reviewer when conflicts could not be resolved. Reference lists of excluded full text papers, flagged reviews, and study protocols identified, were also screened for potential papers of interest.

### Data extraction

A data extraction instrument was created by lead author (TJ) guided by the JBI manual for evidence synthesis [47] and piloted with a sample of selected papers by two independent researchers (BF, TJ). Extractions were compared for similarity and refinements made to the extraction tool with input from the research team. A summary table of the qualitative and quantitative data extraction tool is provided in Additional file 3. When data from a study was reported across multiple papers, all were extracted individually to capture each FN construct investigated, to address the unique aims of each paper. Initial extraction was completed within the Covidence review system software by lead author (TJ) with the second reviewer (BF) independently conducting data extraction on approximately 10% of included full text papers to ensure consensus in extraction. As the purpose of a scoping review is to map evidence, few include a critical appraisal step, with the focus of the current review to investigate and map what types of FN constructs could be found and how these were measured across studies, a critical appraisal step was not conducted to assess study design quality [50].

### Data analysis

Papers were grouped for descriptive analysis based on study type to assess distribution of included study characteristics and data collection methods. FN constructs were initially dichotomised as personal or professional, with constructs allocated to thematically appropriate groups based on the content description and sample questions provided in each eligible research paper. Where a description of the construct was not provided, it was placed in a suitable group based on name only.

No formal statistical analysis was conducted to assess trends in different areas of teacher FN-related health and wellbeing such as nutrition knowledge or dietary intake due to the diversity of construct terminology identified across studies. Instead, further descriptive analysis was conducted using the primary study aim to allocate each paper to one of five groups: Teacher Personal FN, Teacher Professional FN, Student FN, Teacher and Student Personal FN, or Other. Final summary tables were transferred from Microsoft Excel spreadsheets into Microsoft Word and simplified for final presentation.

## Results

**Fig. 1:**
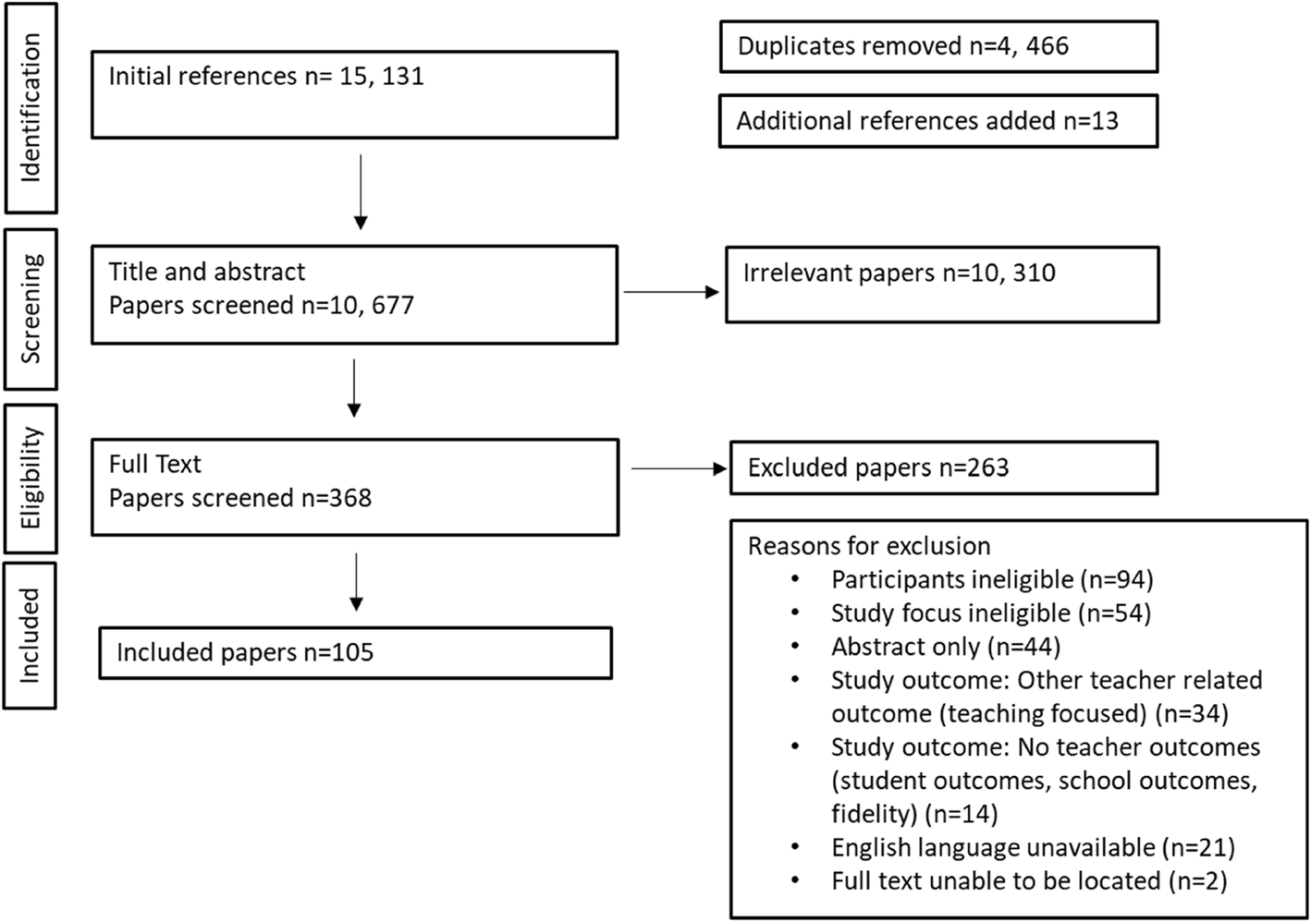
Flow of papers through the different screening phases

Figure 1 illustrates the flow of papers through the different phases with 10,677 unique references screened identifying 368 papers eligible for full text review and a final 105 for inclusion and data extraction. Of the 105 papers, in-service teachers (*n* = 93) were the main participants, with the remaining 12 papers utilising pre-service teachers. Thirty-two papers included teachers with other participants such as guidance teachers’, assistant teachers’, administrative staff, transition teachers and in one instance other health fund members (professions not specified), with a complete summary provided in Additional file 4.

Where some papers specifically mentioned the participant population were teachers, some did not clearly describe them as primary, secondary, or relevant international categories (e.g., junior, middle, high or senior school, and elementary). Where applicable, to clarify this aspect emails (*n* = 24) were sent to authors who were provided a month in which to respond. Thirteen papers where authors’ response was not received were excluded under the “Participants ineligible” exclusion reason as participant population was unable to be confirmed.

**Fig. 2:**
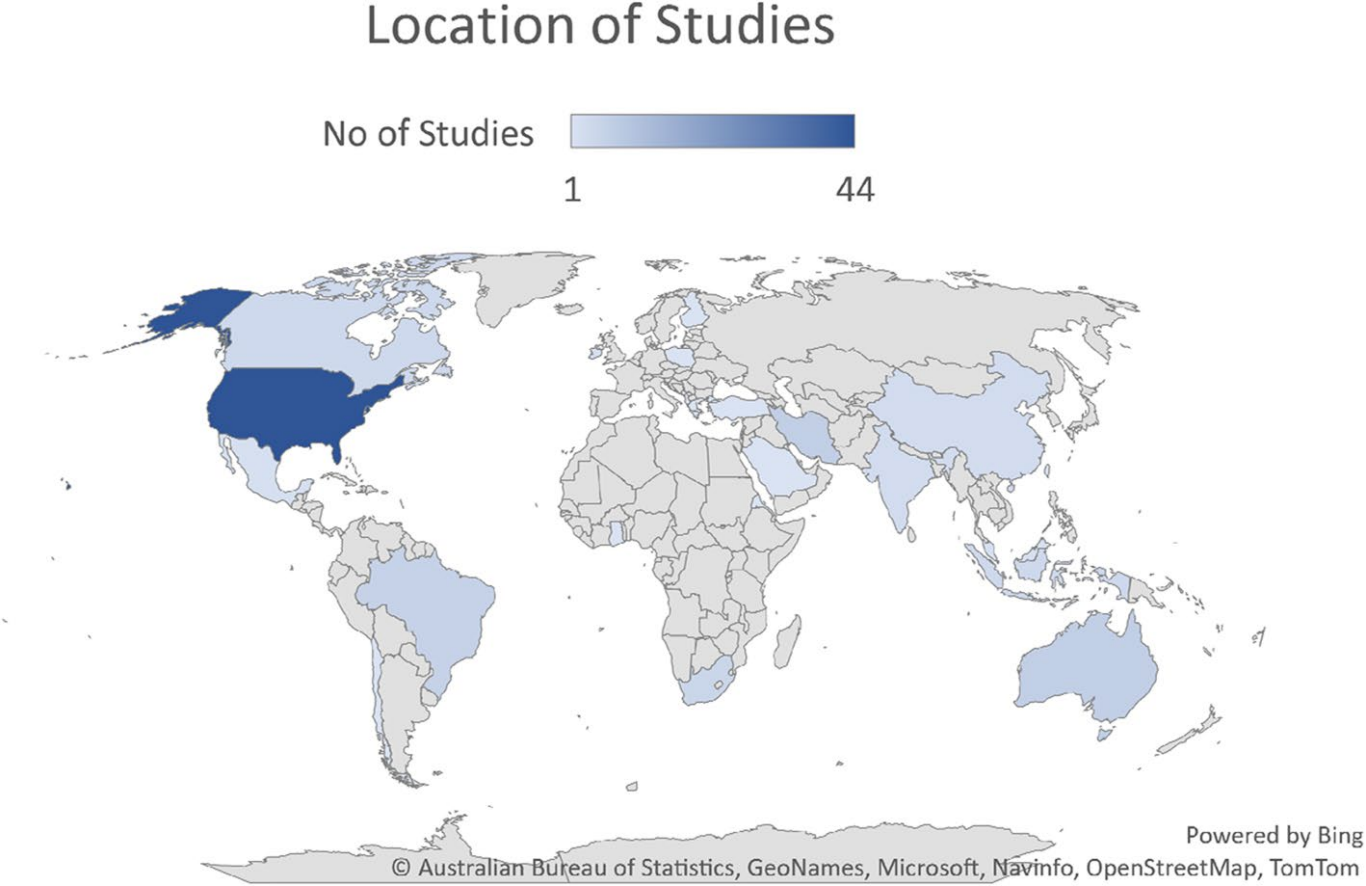
Global distribution of included papers

Figure 2 indicates that studies were predominately from the United States of America (*n* = 44), Australia (*n* = 7), Iran (*n* = 7), Brazil (*n* = 6), South Africa (*n* = 5), Canada (*n* = 4) and Indonesia (*n* = 4). Country of study origin is provided for all included papers in Additional file 4.

Included papers were grouped by study type as defined by Yoong et al. [53] which utilises three groups, descriptive (*n* = 69), intervention (*n* = 35), and measurement (*n* = 1). Included papers being further grouped using the primary study aim into five main groups for analysis of key characteristics and the types of FN constructs used based on the study purpose (Additional file 4). The five groups were labelled, Teacher Personal FN (*n* = 37), Teacher Professional FN (*n* = 30), Student FN (*n* = 11), Teacher and Student Personal FN (*n* = 6) and Other (*n* = 21). Papers that focused on student-related aims favoured incorporating professional teaching FN constructs over personal FN constructs with the main personal FN constructs across these being nutrition knowledge, including food safely knowledge and skills, dietary assessment, and food attitudes. Papers within the ‘Other’ category most often included research focused on diet-disease relationships with teachers acting as a convenience sample with the focus solely on ‘personal FN’ or other health-related and wellbeing covariates or constructs.

Across papers, a wide variety of personal and professional FN constructs were included, with differences noted in how similarly named constructs, such as nutrition knowledge were defined. A summary of all FN constructs identified and thematically grouped based on content descriptions and sample questions is provided in Table 4, with a further breakdown given in Additional files 5 and 6. The four main categories of personal FN: dietary assessment, nutrition knowledge, food or eating habits and behaviours, and nutrition attitudes, are provided in separate tables with content descriptions and sample questions included (Additional file 7).

**Table 4:** Thematically created personal and professional FN construct groups

	**Personal FN constructs *(No’ of papers measuring construct)***	**Professional FN constructs *(No’ of papers measuring construct)***
**Construct groups**	• Dietary assessment (*n* = 41) • Nutrition knowledge (*n* = 33) • Nutrition attitudes (*n* = 15) • Food or eating habits and behaviours (*n* = 23) • Intentions, norms, perceived control, and competence (*n* = 5) • Nutrition practices, resources, and education (*n* = 7) • Food safety practices or knowledge (*n* = 5) • Teacher attitudes and/or eating behaviour at school (*n* = 12) • Dieting status/weight change behaviours (*n* = 9) • Culinary (*n* = 5) • Disordered eating, practices, attitudes, and behaviours (*n* = 5) • Body image (*n* = 7)	• Classroom practices and role modelling (*n* = 15) • School practices, attitudes, and beliefs (*n* = 18) • Education self-efficacy (*n* = 11) • Education intentions (*n* = 3) • Professional development/or resources used (*n* = 5) • Student focused knowledge (*n* = 6) • Barriers to teaching nutrition (*n* = 3) • Teaching characteristics and/or fidelity focus (*n* = 5)

Of the 105 papers, 66 captured one personal FN construct, with dietary assessment (*n* = 31), followed by, nutrition knowledge (*n* = 12) and food or eating habits and behaviours (*n* = 8) being the most prominent, (body mass index and waist circumference were not included as a construct in this description). The remaining 38 papers captured two or more constructs, with different combinations represented including knowledge, attitudes [54], and behaviour or practices [55, 56]; skills, knowledge, practices [57] and behaviour or attitudes and practices [58] as a few examples. Thematic instead of a definitive analysis breakdown was employed to group the most common constructs, due to the lack of consistent terminology, especially in how similarly named constructs were defined across included references. Results of this thematic analysis have been summarised and outlined below, broken down to explore the most common constructs observed across the three main categories of Personal FN, Professional FN and Other-related Health and Wellbeing constructs or covariates.

### Personal food and nutrition

Dietary assessment (*n* = 41) and nutrition knowledge (*n* = 33) were the two most utilised constructs across papers, followed by food or eating habits and behaviours (*n* = 23) and nutrition attitudes (*n* = 15). Additional file 7 provides a summary of the four main construct groups identified using the term provided in the paper of origin to demonstrate the diversity of terminology and ways in which constructs have been defined. Culinary FN constructs were identified across five included papers [59–63], or included as an element within another construct [64] and are summarised in the “Culinary” section of Additional file 5.

#### Dietary assessment

Dietary assessment construct terms included dietary intake (*n* = 36, Additional file 7), dietary habits (*n* = 1) [65], behaviours (*n* = 1) [54], nutrition practice (*n* = 1) [66] and nutrition patterns (*n* = 1) [67], with additional dietary pattern analysis or dietary quality scores being calculated (*n* = 3) [68–70]. Papers measuring dietary intake varied in methodology, using food frequency questionnaires (FFQ) (*n* = 18) [69–86], fruit and vegetable screeners (*n* = 6) [87–92], fat screeners (*n* = 2) [37, 43], dietitian conducted 24-h recalls (*n* = 3) [93–95], automatic 24-h recalls (*n* = 3) [68, 96, 97], a one week food diary (*n* = 1) [98], short food frequency measure (*n* = 1) [99] or brief questions to measure food frequency across select food groups (*n* = 2) [60, 100].

#### Nutrition knowledge

Nutrition knowledge was measured across 33 papers (Additional file 7), with 15 using the construct term ‘Nutrition knowledge’ with various FN-focused questions observed, including local dietary guideline recommendations (*n* = 8) [63, 71, 101–106], nutrient content and/or functions (*n* = 7) [56, 63, 71, 77, 104, 105, 107], diet-disease relationship (*n* = 4) [63, 104, 106, 108], food safety and/or hygiene (*n* = 5) [71, 77, 106–108]. Seventeen additional papers included nutrition knowledge under different construct terms or scores including nutrition literacy [109], knowledge of nutrition score and a knowledge of nutrients functions score [66], healthy food choices knowledge score [110], or combined in a multi-faceted construct that included nutrition knowledge along with attitudes and practices questions [111]. Where constructs were identified separately as knowledge or attitudes they have been allocated to each specific thematic category. Those that did not provide clearly separated constructs and were included together are identified in the thematic analysis of only one category based on the content description provided. While some papers within this category included food safety questions (*n* = 4) [55, 77, 106, 108], those that focused solely on food safety knowledge, practices or skills were grouped separately under food safety practises or knowledge (*n* = 5) [57, 112–115]. Ten papers provided sample questions [2, 64, 101, 102, 106, 110, 116–119], with two papers stating nutrition knowledge was assessed, with another providing two nutrition knowledge scores, but none of these provided a description, tool reference or sample question to identify how nutrition knowledge was defined [54, 66, 120].

#### Food or eating habits and behaviour, and nutrition attitudes

Within the food or eating habits and behaviours category a range of construct terms were identified across the 23 included papers (Additional file 7). The most common were eating habits, including frequency of meals or snacks consumed daily (*n* = 4) [60, 61, 71, 121] or meal skipping practices (*n* = 2) [66, 122]. Six papers used or included an adapted version of the Personal Health Index [43] which has six single item questions around teacher health perceptions, level of satisfaction with their eating habits and regularity of consuming recommended fruit and vegetable serves [2, 37, 43, 46, 99, 123]. Self-regulation of diet was assessed in two papers utilising the Treatment Self-Regulations Questionnaire for Diet that incorporates the self-determination theory approach [124, 125]. One study indicated evaluating dietary and hygienic habits without description of construct content [126]. Of the 15 papers (Additional file 7) that included a nutrition attitudes construct, two included no description of the content covered [55, 127], three papers included attitudes with practice or behaviours [56, 58, 103] with one paper capturing two attitude scores including food value orientation and food waste attitudes [58].

#### Body image, disordered eating, dieting status and weight change behaviours

Body image (*n* = 7), disordered eating (*n* = 5), dieting status and weight change behaviours (*n* = 9), (See Additional file 5) were not included within the food or eating habits and behaviour, or nutrition attitudes categories, remaining as separate construct categories due to the specificity of the health and psychological behaviours being explored.

#### Culinary

Five papers measured culinary focused constructs [59–63] with one paper including more than one construct (e.g., cooking attitudes, frequency of home meal preparation and average time spent preparing a meal) [59]. One paper [60] used two single item questions to identify basic cookery practices in relation to health, including use of salt in cooking and type of fats and oils used, with two further studies measuring confidence and self-efficacy in conducting culinary practices or an individual’s level of food literacy [61, 62]. Another paper included two single item questions to identify which participants were responsible for home meal preparation and frequency of meal preparation [63]. Finally, to demonstrate the diversity of construct terminology, one paper not counted as including a culinary construct did identify single item questions with a culinary focus, within their nutrition knowledge and behaviour construct that measured a participant’s ability to identify healthy cooking practices (e.g., steaming) [64].

### Professional food and nutrition

Of 42 papers measuring some aspect of professional FN (Additional file 6), all had a Teacher Professional FN primary aim apart from six papers that identified a student FN [128–133] primary aim (Additional file 4). The school practices attitudes and beliefs category (*n* = 18, Additional file 6) was the largest Teacher Professional FN category observed among included papers, with one paper identified multiple times using three constructs to collect data on teacher perceptions of school wide food practices, beliefs regarding the school-food environment and food-related school policy [134]. Another paper measured two aspects of school practices, attitudes, and beliefs, being, the school food environment and teachers’ perceptions of the importance of aspects of food literacy [62]. The classroom practices and role modelling category were the next largest (*n* = 15), followed by, nutrition education self-efficacy (*n* = 11). These were also the most frequently observed construct groups with common tools used across included papers including the Classroom Food Practices construct, the School Food Environment Index [43] and the Nutrition Teaching Self-Efficacy Measure [44].

### Other-related health and wellbeing covariates and constructs

Physical activity and/or exercise, including self-regulation of these was the most common covariate or construct included across 43 of the included papers, followed by smoking and/or smoking status and tobacco use (*n* = 23), alcohol intake (*n* = 11), and sleep (*n* = 5). With mental health and wellbeing measured in 11 [60, 68, 90–92, 122, 135–139] of the 105 papers, including perceived stress [122] and perceived occupational stress [68]. Three papers utilised a personal health assessment to report work related aspects of job performance, along with life satisfaction, and related mental health outcomes such as depression, stress, and loneliness [90–92]. One study included an aspect of mindfulness [137] with two papers using different measures to assess an Individual Lifestyle Profile and the Assessment Scale of the Quality of Life at Work Perceived by Primary and Secondary School Physical Education Teachers, which included aspects of work conditions and opportunities, job autonomy and social integration in the workplace [135, 136]. Perceived organisational commitment to employee health was measured in one study [139]. Frequency of practicing a collection of five healthy habits, including mental health was included in a larger measure of one paper [138], with a final paper using two single item questions to evaluate if participants had any organic or psychiatric diseases in a yes and no style question format [60].

### Characteristics of the tools used to collect food and nutrition data

Table 5 demonstrates the distribution of data collection methods used across included papers with questionnaires (*n* = 99) being the predominate data collection method, with participants self-reporting responses in paper-based or digital format. Five papers used researcher assistance to complete questionnaires [126, 131] with three being a part of the one study [72–74]. Few papers that used questionnaires listed the average completion time, with those that did, indicating completion took between 10–20 minutes [60, 88, 103], two others mentioned either a longer completion time of 45 minutes [139] or shorter, approximately eight minutes [92]. Other data collection methods included anthropometric data (*n* = 51), followed by health-related data, which included blood pressure or fasting blood samples (*n* = 15). Physical assessment was measured in four papers with three papers, from the same study, using accelerometers with participants [93–95]. Linkage data used in eight papers provided data from state mortality files with qualitative data collected in fifteen papers using interview methods of data collection (*n* = 11) or focus groups (*n* = 4). Validation and reliability testing methods were reviewed for the data collection methods used across included papers with descriptions of psychometric testing often unclear, missing or a reference provided to indicate additional information on tool development and/or testing was reported elsewhere. Of the 105 papers reviewed only 22 papers included a clear description of validation methods used, with 13 providing an explanation of the reliability testing conducted. Further analysis of psychometric testing of data collection methods reviewed was beyond the scope of this current review (Additional file 4).

**Table 5:** Data collection methods (breakdown provided by study type)

	**Questionnaire**	**Qualitative**			**Health-Related**			**Anthropometric**		
**Study Type**/***Data collection method***	***Questionnaire***	***Observations***	***Interview***	***Focus Group***	***Blood samples or Blood pressure examination***	***Physical Assessment***	***Linkage Data***	***Anthropometric Self-reported***	***Anthropometric Researcher Measured***	***Anthropometric Not Specified***
**Descriptive**	64	0	8	3	10	4	8	18	18	4
**Intervention**	34	2	3	1	5	0	0	5	3	2
**Measurement**	1	0	0	0	0	0	0	0	1	0
Total	99	2	11	4	15	4	8	23	22	6
References	Complete reference list and data collection method summary in Additional file 4	[87, 129]	[89, 100, 102, 112, 117, 119, 120, 130, 140–142]	[61, 112, 133, 143]	[68, 70, 72–75, 92, 94, 96, 100, 103, 111, 117, 122, 137]	[93–95, 100]	[69, 80–86]	[2, 46, 59, 63, 67, 71, 80–86, 88, 90, 97, 106, 132, 143–147]	[60, 65, 66, 68, 70, 72–75, 78, 79, 93–95, 100, 103, 111, 117, 121, 122, 137, 139]	[42, 69, 92, 96, 98, 148]

## Discussion

The current scoping review has summarised study characteristics and data collection methods used to measure FN-related health and wellbeing in teachers. The aim was to examine the types and range of FN constructs that have been used to date, particularly in reporting personal FN constructs. The results indicate that FN constructs have been reported across a range of study designs with diverse aims and disciplines, to measure data on personal and professional aspects of FN-related health and wellbeing in teachers. The major finding is that the constructs used in research to date are highly variable and lack consistency in construct terminology. Dietary habits were one construct appearing in two included papers [60, 65], with another four including dietary habits examining eating habits [135, 136], eating behaviours [122] or hygienic behaviours [126], yet these were placed in three different construct groups during thematic analysis based on content variations within the papers. The three construct groups dietary habits appeared in were dietary assessment [65], food or eating habits and behaviours [122, 126, 135, 136], and culinary [60]. Given that papers have been published internationally, some diversity in construct content is to be expected due to global differences in food based dietary guidelines, cultural food preferences and practices, and local food sources. However, the diversity extends beyond these expected variations with this clearly demonstrated in detail across Additional files 5,  6 and 7 where each construct identified is listed by the names given or described across included papers. Therefore, to assess those most frequently used and to identify common themes and gaps, thematic analysis was conducted. Where possible the results of common construct themes are discussed in relation to the DONE framework as a guide to the variety of determinates that impact healthy eating as well as their potential modifiability, population level of effect and suggested research priorities [10, 11].

The DONE framework identifies 51 determinate groups, that contribute to or influence FN choices and healthy eating actions of individuals [10, 11]. These determinates are placed into four categories of individual, interpersonal, environmental and policy, and have been rated within this framework based on their modifiability, population level of effect and the relationship strength between the two. Determinates with a higher ranking in all three areas are then listed in order of potential research priority.

### Measuring teacher personal food and nutrition-related health and wellbeing, the determinates, and correlates to consider

#### Dietary assessment

Results of the current review indicate dietary assessment was the most used construct across all studies, especially within papers that included only one personal FN construct. Diet quality is an established correlate of health-related outcomes, used globally to assess dietary risks regarding morbidity and mortality [149, 150] and used across a range of study designs. The use of FFQ or dietary screeners, like those used within included papers (Additional file 7), make the assessment of dietary intake practical and efficient to incorporate within research studies, with brief dietary screeners providing indicators of diet quality while reducing researcher and participant burden [34].

Diet quality indexes have recently been used as a diet-related health indicator in mental health and wellbeing interventions [14]. However, despite the recognition that teacher populations globally experience high levels of stress and burnout [29, 31], especially with additional pressures observed throughout the COVID-19 pandemic [151], only 11 of the current included review papers utilised a measure of mental health and/or wellbeing such as quality of life [135, 136] or perceived stress [152]. It is important that future research in teacher FN-related health and wellbeing includes investigation or consideration of the impact of key mental health-related factors such as stress, anxiety and/or burnout on teacher diet quality and other FN-related factors.

#### Nutrition knowledge

Despite nutrition knowledge being acknowledged within the DONE framework as a determinate of healthy eating and being potentially modifiable [10, 11], it only has a weak positive correlation with overall health and wellbeing [153]*.* Nutrition knowledge can be modified by education programs, which likely explains its frequent inclusion within included papers of this review and was the second most utilised construct observed [55, 102, 108, 154]. However, nutrition self-efficacy, dietary knowledge, and food knowledge all appear as stronger research priorities within the DONE framework determinate category that nutrition knowledge is grouped with. Nutrition education self-efficacy was captured as a professional FN construct in five of the included papers, with other papers exploring aspects of self-efficacy [46, 101, 102, 123, 129] and confidence to teach FN curriculum [105] or intervention materials [87]. Only one paper developed a specific measure to assessed personal food literacy self-efficacy [62], with others exploring aspects of healthy eating confidence by single items questions [105]. Overall, nutrition knowledge has a weak positive correlation with dietary intake [153] with other constructs that incorporate aspects of nutrition knowledge, nutrition self-efficacy and dietary knowledge such as food agency [13] and food skills confidence [12] identified as stronger correlates of health-related outcomes, including diet quality. Therefore, future research that investigates the connections between FN factors and health and wellbeing outcomes of teachers should consider incorporating constructs that measure aspects of nutrition knowledge yet have potentially stronger relationships with FN-related health and wellbeing outcomes.

#### Food or eating habits and behaviour, and nutrition attitudes

Within the DONE framework the Individual/psychological section includes nine determinate categories, of which health cognitions, followed by food habits, food knowledge, skills and abilities, and food beliefs are the top research priories in relation to healthy eating practices of individuals and populations [10, 11]. Within the current review food and eating habits and behaviour and nutrition attitudes were the third and fourth most commonly measured constructs in teacher participants. Six papers within the current review included a version of the Personal Health Index [43] or used an alternate measure to assess perceived health or health status [67, 90–92, 144]. Within the health cognitions determinate category of the DONE framework perceived health ranks the lowest, with health consciousness, health concerns, healthy eating motivation and healthy eating intentions listed as determinants with higher research priorities, potentially higher modifiability, and/or population level of effect [10, 11]. Healthy eating motivations and intentions were minimally covered across included papers [42, 110] and could present a potential area of further exploration in future teacher FN focused research.

#### Body image, disordered eating, dieting status and weight change behaviours

Within the current review eight papers included at least one construct related to body image, disordered eating, dieting status and/or weight change behaviours with three focused on teacher professional responsibilities. The remaining studies were classified within the current review as papers with personal aims, however, the focus did not include consideration of how these constructs may influence teacher overall health and wellbeing. Weight control cognitions and behaviours is noted within the individual/psychological level/category of the DONE framework, however, when considering the potential modifiability, population level of effect and overall research priority ranking only weigh loss intentions is included within the top few below nutrition knowledge, dietary knowledge, health consciousness and cooking skills [10, 11].

#### Culinary

Of the five papers that included a culinary construct, two measured participant food involvement or level of meal preparation participation which is included within the food beliefs determinate category of the DONE framework [10, 11]. However, none of these measured cooking skills which is a factor identified, within the food knowledge, skills, and abilities determinate group. With culinary inclusive interventions exploring the use of cooking skills [38, 155] in connection with dietary intake, and cooking confidence as a potential correlate of wellbeing outcomes in adults, not teacher participants [14–16], future FN research in teachers might consider further exploration of these two culinary constructs.

### Other-related health and wellbeing constructs and covariates

Within the related health behaviours determinate category physical activity level is identified, along with alcohol consumption as covariates of health and diet related outcomes. Sleep was measured in five of the 105 included studies often as a single item question to assess sleep quality or quantity. While sleep ranks relatively low as a research priority connected to healthy eating within the DONE framework [10], it is acknowledged as a contributor and potential correlate of mental health and wellbeing outcomes [156]. Considering these findings, future teacher FN-related research should consider the covariates or other health related constructs that may contribute to or influence both the FN and related-health and wellbeing of teachers.

### Measuring teacher professional food and nutrition, and student impact

The focus of the current review was to evaluate how and where personal FN constructs have been reported in research assessing FN-related health and wellbeing in teachers. This is an important factor to consider given the additional FN roles and responsibilities teachers hold and their potential to impact the school environment and students within their care [1, 157]. Of 42 papers that included one or more professional FN constructs, eight categories were created to collate the observed constructs. While variety in terminology was also an issue that impeded overall analysis, it was observed that what is being assessed in relation to teacher professional FN roles was better defined than teacher personal FN constructs. Although these findings should be interpreted with caution as only studies that included at least one personal FN factors were considered for inclusion in this review and used in the current analysis.

#### Classroom food related practices

Food rewards are commonly used by teachers globally as a classroom incentive, with energy-dense, nutrient-poor food items and beverages such as candy, pizza and sugar sweetened beverages most often selected over the preferred healthier options [158]. Within the DONE framework, food used as an incentive is identified within the parental feeding styles and parental behaviours determinate categories and is acknowledge as being impactful to healthy eating outcomes of children [10, 11]. Teachers’ have been viewed similarly as gatekeepers of influence within the school environment, through the provision and use of food rewards, measured in many of the papers in the current review within nutrition-related classroom practices, food-related classroom practices, or in the case of pre-service teachers, their future classroom intentions [37, 46]. Of the 10 papers that measured this determinate, half explored the connection between a teacher’s personal FN factors and their use of food rewards [2, 37, 43, 46, 97]. While different methods were used to explore this across included papers, personal FN factors such as diet quality, nutrition knowledge and personal health perceptions were noted as indicative of use of food rewards and level of teacher engagement in their professional FN roles. Of included papers, one reported that pre-service teachers with lower personal health perceptions and higher BMI were more likely to report using food rewards [46] with another identifying that diet quality within their study was positively correlated with better classroom nutrition practices [97]. Teachers of subject areas who most frequently receive some nutrition education such as physical education and consumer science (e.g., home sciences or home economics) were noted in one paper to be more likely to role model healthy habits and less likely to provide low nutritive food rewards [43]. Future studies should consider this connection in acknowledging how a teachers’ FN-related health and wellbeing can potentially impact student-related health and wellbeing.

### Teacher personal and professional food and nutrition

The professional FN roles and responsibilities of teachers have been the focus of research to date, with personal FN being explored more frequently in the last two decades. However, the two should not be considered in isolation with this review highlighting the many personal FN determinates such as nutrition knowledge [46, 108], food related practices [43, 90, 116], and beliefs about healthy nutrition [42], that may impact a teacher’s ability to be positive FN role models, health promoters, gate keepers and FN educators. Teachers need to be supported to achieve and maintain good personal FN practices to better support them in healthfully approaching their professional FN roles and general teacher practices towards achieving positive health promoting school environments.

### Data collection methods

Questionnaires were the main method of assessing many different constructs (*n* = 99). Self-reported questionnaires have lower participant burden and can incorporate multiple constructs of interest to address a wide range of study designs. While this method can be prone to participant completion error or bias, when conducting research with teacher participants reducing teacher participation time is a key factor for this population group who are usually time poor. Reducing participant burden as a strategy to increase study participation using questionnaires with close-ended responses may therefore be an effective way to optimise questionnaire completion. Within the current review descriptions of psychometric testing conducted on included data collection methods was limited and often unclear, with complete descriptions of items and scales used within questionnaires rarely included. This lack of validation and reliability descriptions and clear outline of the items or scales included within questionnaires makes comparison between tools challenging and limits other researchers in utilising these tools in future research.

### Implications for research

The current scoping review mapped the research examining how teacher FN-related health and wellbeing has been studied across a wide range of study designs, and the main FN constructs used to assess it identifying key gaps. Results can be used to guide future school and teacher focused research, that incorporates teacher’s personal and professional FN constructs and their impact on individual teacher-related health and wellbeing.

### Strengths and limitations

The current review study is the first to comprehensively investigate where and how personal FN constructs and related health and wellbeing factors have been used or measured across education and health research that included teacher participants. Recommended guidelines for scoping review [47] methodology were observed at all stages and allowed a wide net to be cast, gathering papers from various fields of research. While this added to the diversity of study designs, themes and gaps mapped, it limited traditional data synthesis due to the heterogeneity of construct terminology identified. While all included papers were published in peer reviewed journals a formal critical appraisal was not conducted.

Where papers did not provide a clear description of teacher FN component(s) in the abstract, potentially relevant papers may have been excluded in screening phase. Hence, although many studies are included, results should be interpreted with caution. Additionally, studies that indicated a teacher FN component, but did not provide a clear description within the methods were excluded at full text screening for lack of detail.

While thematic analysis provided a broad overview of the common themes and gaps, it is acknowledged that the strength of the current review is the mapping of the definitions, descriptions, and sample questions detailing FN-related constructs provided in the included papers. Therefore, where limited description was provided, these constructs may have been incorrectly placed within a construct category. However, this further highlights the need for more clarity and detailed descriptions of constructs used within teacher interventions and the possible sharing or inclusion of sample questions, to ensure research is represented as intended in future studies and the need for standardisation of construct terminology.

## Conclusion

This is the first review to map where and how teacher personal FN have been reported across international research including student and school focused papers. While facets of teacher FN have been studied across a wide range of research areas, the lack of validated tools or clearly defined evidence-based FN constructs used in research to date makes comparisons or assessment of teacher population personal FN status challenging. Future research is needed to address these gaps.

### Supplementary Information

**Additional file 1.** PRISMA-Scr Checklist.

**Additional file 2.** Search strategy by Database.

**Additional file 3.** Overview of Data Extraction Table.

**Additional file 4.** Primary Aims Summary Table (All Included Studies).

**Additional file 5.** Personal FN-Summary of Constructs Observed.

**Additional file 6.** Professional FN-Summary of Constructs Observed.

**Additional file 7.** Main Constructs.

## Data Availability

Not available.

## Abbreviations

FN: Food and Nutrition
DONE: Determinates Of Nutrition and Eating
PRISMA-Scr: Preferred Reporting Items for Systematic Reviews and Meta-Analysis extension for Scoping Reviews
PCC: Population Concept Context
