# Supplementary material for: Addressing schoolteacher food and nutrition-related health and wellbeing: a scoping review of the food and nutrition constructs used across current research
Source: Int J Behav Nutr Phys Act. 2023 Sep 12;20:108. doi: 10.1186/s12966-023-01502-5 (PMC10498614; doi:10.1186/s12966-023-01502-5)
Supplement: Supplementary file 2 — Additional file 2. Search strategy by Database. [file 12966_2023_1502_MOESM2_ESM.docx]

| **PCC Elements** | **Search Terms** | **Fields/Actions** |
| --- | --- | --- |
| ***CINAHL*** | | |
| S1  Context | (MH "Schools") OR (MH "Schools, Secondary") OR (MH "Schools, Middle") OR (MH "Schools, Elementary") | Expanders - Apply equivalent subjects  Search modes - Boolean/Phrase |
| S2  Population | ( ("early career” OR inservice OR “in service” OR preservice OR “pre service” OR prospective OR student*) n3 teacher*) OR ( (primary OR elementary OR headstart OR “early childhood” OR secondary OR high OR middle OR school*) n3 (teacher* OR schoolteacher* OR educator*)) | Expanders - Apply equivalent subjects  Search modes - Boolean/Phrase |
| S3 | S1 OR S2 | Expanders - Apply equivalent subjects  Search modes - Boolean/Phrase |
| S4  Concept | ( (food* OR nutri* OR diet* OR cook* OR eat*) n3 (belief* OR attitude* OR habit* OR quality* OR literac* OR health OR educat* OR program OR train* OR wellbeing OR “well being” OR culinary OR curricul* OR knowledge OR status OR polic* OR skill* OR agency OR pedagogy OR behavio#r* OR practic* OR experience* OR motivat* OR “self efficacy” OR “self perception” OR classroom* OR environment OR model* OR advocat*) ) | Expanders - Apply equivalent subjects  Search modes - Boolean/Phrase |
| S7 | S3 AND S4 | Limiters - Peer Reviewed  Expanders - Apply equivalent subjects  Search modes - Boolean/Phrase |
| ***Embase*** | | |
| 1  Context | Schools/ |  |
| 2  Population | ((primary or elementary or headstart or “early childhood” or secondary or high or middle or school) adj3 (teacher* or schoolteacher* or educator*)).mp. |  |
| 3  Population | (("early career" or inservice or "in service" or "pre service" or preservice or prospective or student) adj3 teacher*).mp. |  |
| 4  Concept | ((food* or nutri* or diet* or cook* or eat*) adj3 (belief* or attitude* or habit* or quality* or literac* or health or educat* or program or train* or wellbeing or "well being" or culinary or curricul* or knowledge or status or polic* or skill* or agency or pedagogy or behavio?r* or practic* or experience* or motivat* or "self efficacy" or "self perception" or classroom* or environment or model* or advocat*)).mp. |  |
| 5 | 1 or 2 or 3 |  |
| 6 | 4 and 5 |  |
| 7 | limit 6 to english |  |
| ***Medline*** | | |
| 1 | Schools/ | Key word/title/abstract |
| 2 | ((primary or elementary or headstart or “early childhood” or secondary or high or middle or school) adj3 (teacher* or schoolteacher* or educator*)).mp. | Key word/title/abstract |
| 3 | (("early career" or inservice or "in service" or "pre service" or preservice or prospective or student) adj3 teacher*).mp. | Key word/title/abstract |
| 4 | ((food* or nutri* or diet* or cook* or eat*) adj3 (belief* or attitude* or habit* or quality* or literac* or health or educat* or program or train* or wellbeing or "well being" or culinary or curricul* or knowledge or status or polic* or skill* or agency or pedagogy or behavio?r* or practic* or experience* or motivat* or "self efficacy" or "self perception" or classroom* or environment or model* or advocat*)).mp. | Key word/title/abstract |
| 5 | 1 or 2 or 3 |  |
| 6 | 4 and 5 |  |
| 7 | limit 6 to english |  |
| ***PsycInfo*** |  |  |
| 1 | Schools/ |  |
| 2 | ((primary or elementary or headstart or “early childhood” or secondary or high or middle or school) adj3 (teacher* or schoolteacher* or educator*)).mp. |  |
| 3 | (("early career" or inservice or "in service" or "pre service" or preservice or prospective or student) adj3 teacher*).mp. |  |
| 4 | ((food* or nutri* or diet* or cook* or eat*) adj3 (belief* or attitude* or habit* or quality* or literac* or health or educat* or program or train* or wellbeing or "well being" or culinary or curricul* or knowledge or status or polic* or skill* or agency or pedagogy or behavio?r* or practic* or experience* or motivat* or "self efficacy" or "self perception" or classroom* or environment or model* or advocat*)).mp. |  |
| 5 | 1 or 2 or 3 |  |
| 6 | 4 and 5 |  |
| ***Scopus*** | | |
|  | ( TITLE ( ( *schools*  OR  ( ( *teacher**  OR  *schoolteacher**  OR  *educator** )  W/3  ( *primary*  OR  *elementary*  OR  *headstart*  OR  *"early childhood"*  OR  *secondary*  OR  *high**  OR  *middle**  OR  *school** ) )  OR  ( ( *"early career"*  OR  *inservice*  OR  *"inservice"*  OR  *"preservice"*  OR  *preservice*  OR  *prospective*  OR  *student* )  W/3  *teacher** ) ) )  AND  TITLE ( ( ( *food**  OR  *nutriti**  OR  *diet**  OR  *cook**  OR  *eat** )  W/3  ( *role**  OR  *belief**  OR  *attitude**  OR  *habit**  OR  *quality**  OR  *literac**  OR  *health*  OR  *educat**  OR  *prog**  OR  *train**  OR  *wellbeing*  OR  *"well being"*  OR  *culinary*  OR  *curricul**  OR  *knowledge*  OR  *status*  OR  *polic**  OR  *skill**  OR  *agency*  OR  *pedagog**  OR  *behavio?r**  OR  *practice**  OR  *experience**  OR  *motivation**  OR  *"self efficacy"*  OR  *"self perception"*  OR  *classrooom**  OR  *environment**  OR  *model**  OR  *advocate** ) ) ) ) | Article and Review  English Language  Article title only |
| ***Eric via ProQuest*** | | |
|  | ab((schools OR (("early career" OR inservice OR " in service" OR "pre service" OR preservice OR prospective OR student) NEAR/3 teacher*) OR ((primary OR elementary OR headstart OR "early childhood" OR secondary OR high OR middle OR school) NEAR/3 (teacher* OR schoolteacher* OR educator*)))) AND ab(((food* OR nutri* OR diet* OR cook* OR eat*) NEAR/3 (belief* OR attitude* OR habit* OR quality* OR literac* OR health OR educat* OR program OR train* OR wellbeing OR "well being" OR culinary OR curricul* OR knowledge OR status OR polic* OR skill* OR agency OR pedagogy OR behavio?r* OR practic* OR experience* OR motivat* OR "self efficacy" OR "self perception" OR classroom* OR environment OR model* OR advocat*))) AND (la.exact("ENG") AND PEER(yes)) | Abstract  English language  Peer-reviewed |
