# Supplementary material for: Addressing schoolteacher food and nutrition-related health and wellbeing: a scoping review of the food and nutrition constructs used across current research
Source: Int J Behav Nutr Phys Act. 2023 Sep 12;20:108. doi: 10.1186/s12966-023-01502-5 (PMC10498614; doi:10.1186/s12966-023-01502-5)
Supplement: Supplementary file 3 — Additional file 3. Overview of Data Extraction Table. [file 12966_2023_1502_MOESM3_ESM.docx]

| **Data extracted** | **Description** |
| --- | --- |
| Study ID |  |
| Title |  |
| Publication Date |  |
| Study Period |  |
| Authors |  |
| Country | In which the study was conducted |
| Data type collected | Qualitative, Quantitative, Both |
| Data collection method(s) |  |
| Self-reported measures or other |  |
| Study Type | As defined by …Description, Intervention, Measurement |
| Study design |  |
| Was it part of a larger study   - Which study |  |
| Aims   - Primary - Secondary |  |
| Primary Purpose   - - PP-Assess/evaluate     - What   - PP-Intervention (not training)     - Description     - Intervention outcomes |  |
| Population description   - Teacher level - Teacher type - Inclusion criteria - Exclusion criteria - Total number of participants (teachers only) |  |
| Measure   - Number of constructs - Constructs - Number of items/questions - Time taken to complete - Validity (Y/N) - Reliability (Y/N) - Type of validity or reliability (Face, Content, Criterion, Construct, test-re-test) - Description of measure - Measure reference |  |
| Food and nutrition constructs investigated   - Personal - Professional |  |
| Other health and lifestyle covariates or constructs measured beyond food and nutrition |  |
| Training provided (Y/N)   - - Training focus   - Length   - Format   - Resources provided (Y/N)   - Resources description   - Support provided (Y/N)   - Support description   - Training description |  |
| Research outcomes  Personal   - BMI status - Dietary - Chronic disease - Culinary - Food and nutrition knowledge - Other   Professional   - Classroom practices - Nutrition education - Role model - Health promoter - Other |  |
| Notes   - Study impact - Research gaps noted |  |
| Combined data |  |
| Questions provided | If questionnaire used, where questions provided |

Legend: Text in grey was extracted but not selected for discussion in the final scoping review.
