# Supplementary material for: Addressing schoolteacher food and nutrition-related health and wellbeing: a scoping review of the food and nutrition constructs used across current research
Source: Int J Behav Nutr Phys Act. 2023 Sep 12;20:108. doi: 10.1186/s12966-023-01502-5 (PMC10498614; doi:10.1186/s12966-023-01502-5)
Supplement: Supplementary file 4 — Additional file 4. Primary Aims Summary Table (All Included Studies). [file 12966_2023_1502_MOESM4_ESM.docx]

**Table 1** Teacher Personal FN Focused Primary Study Aim

| **Source (Author, Year, Country)** | **Primary Study Aim^**  **Teacher Personal FN** | **Study (Type, Design used, Data type collected)** | **Teachers (Type, Level taught)** | **Personal Food and Nutrition Factors Explored** | **Professional Food and Nutrition Factors Explored** | **Other Health and Wellbeing Factors Explored** |
| --- | --- | --- | --- | --- | --- | --- |
| Al-Gelban 2008, Saudi Arabia^1^ | To assess the dietary habits and exercise practices of students at teachers' training college Abha, Kingdom of Saudi Arabia. | Descriptive,  Cross-sectional. *Questionnaire, Anthropometric* | Inservice,  Primary | - Dietary intake - Body mass index - Dietary habits - Eating habits |  | - Exercise practices - Current health conditions |
| Ates 2019, Turkey^2^ | To examine the effects of elementary school teachers' psychological factors on their behavioural intentions for healthy nutrition in their daily life within an extended version of the theory of planned behaviour. | Descriptive,  Correlation study.  *Questionnaire, Anthropometric* | Inservice,  Elementary | - Body mass index - Aspects of Healthy Eating explored through:   - - Subjective norm     - Attitudes     - Perceived behaviour control     - Intention     - Personal norms     - Self-identity     - Behaviour |  |  |
| Both 2016, Brazil^3^ | Aims to assess the relationship between multiple job holdings and the wellbeing of physical education teachers from Southern Brazil. | Descriptive,  Exploratory descriptive and Cross-sectional. *Questionnaire*  R | Inservice,  Elementary, High school | - Dietary/eating habits |  | - Physical activity - Preventative behaviours - Relationships - Stress control - Work related measures and wellbeing |
| Both 2017, Brazil^4^ | To evaluate the relation between gender and PE teachers' wellbeing in southern Brazil, considering the socio-environmental (job) and individual (lifestyle) parameters. | Descriptive,  Descriptive cross sectional.  *Questionnaire*  VR | Inservice,  Elementary, High school | - Dietary/eating habits |  | - Quality of life/health rating - Physical activity - Stress control - Job satisfaction/workplace factors |
| Chen 2010, Taiwan^5^ | To investigate the effects of implementing health promoting schools’ programmes on teachers’ nutrition knowledge and diets. | Intervention, Case control. *Questionnaire, Anthropometric*  VR | Inservice,  Elementary | - Dietary intake - Body mass index - Nutrition knowledge - Dietary behaviour - Weight perception | - Food and nutrition focused professional development completed in the past three years |  |
| Dalais 2014, South Africa^6^ | To investigate not only primary school educators' health status but also their knowledge, perceptions, and behaviour regarding the two most important behavioural aspects of non-communicable disease risk, diet, and physical activity. | Descriptive,  Cross sectional descriptive. *Questionnaire, Anthropometric, Clinical* | Inservice,  Primary | - Body mass index - Waist Circumference - Nutrition knowledge - Nutrition attitudes and behaviours - Perceived weight - Attempts at weight loss |  | - Physical activity - Active participation in sport/exercise - Smoking - Family history of non-communicable diseases - Blood pressure - Non-fasting glucose levels |
| Delfino 2020, Brazil^7^ | To analyse in the same research the relation between sedentary behaviour and breaks in sedentary time at work and leisure with dietary and lifestyle habits in public school teachers, controlled by confounding factors such as sex, age and socioeconomic status. | Descriptive,  Cross-sectional. *Questionnaire* | Inservice,  Secondary | - Dietary habits - Body mass index |  | - Physical activity - Smoking - Sedentary behaviour - Alcohol intake |
| Dunn 2013, United States of America^8^ | To ascertain the effectiveness of a behaviour-change weight management program offered to teachers and state employees in North Carolina. | Intervention, Non-randomised experimental. *Clinical, Questionnaire, Anthropometric* | Inservice*,  Primary, Secondary | - Healthy eating changes: Confidence in ability to eat healthfully - Body mass index |  | - Mindfulness - Physical activity - Weight loss classification and weight throughout intervention - Confidence to be physically active - Blood pressure |
| Frerichs 2016, United States of America^9^ | To investigate the role of the physical environment in shaping healthy eating attitudes and practices among school staff members. | Descriptive,  Mixed methods approach. *Questionnaire, Interview* | Inservice*,  Primary, Elementary | - Dietary intake | - Classroom food practices - School food environment - Role modelling capability and classroom food practices |  |
| Gebretatyos 2020, Eritrea^10^ | Aimed at assessing the effect of health education on healthy nutrition and physical activity among 40-60-year-old female teachers in elementary, junior, and secondary schools of Asmara. | Intervention, Quasi. *Questionnaire*  VR | Inservice,  Elementary, Junior high, Secondary | - Nutrition |  | - Physical activity - Spiritual growth - Interpersonal relations |
| Hartline-Grafton 2010, United States of America ^11^ | Examined the eating patterns of female elementary school personnel and whether these patterns, specifically eating occasion frequency, were associated with energy intake and body mass index. | Descriptive,  Cross-sectional. *Anthropometric, Accelerometer, 24-hour Recall, Questionnaire* | Inservice*,  Elementary | - Dietary intake - Dietary pattern analysis - Body mass index |  | - Physical activity - Tobacco use - Job category |
| Hartline-Grafton 2009, United States of America^12^ | To investigate selected dietary variables and weight status among elementary school personnel. | Descriptive,  Cross-sectional. *Anthropometric, 24-Hour Recall, Questionnaire, Accelerometer* | Inservice*,  Elementary | - Dietary intake - Body mass index |  | - Physical activity - Tobacco use - Job category |
| Hartline-Grafton 2009, United States of America^13^ | Examined the independent simultaneous associations of Energy Density Foods and Energy Density Beverages on energy intake and body mass index in adult women. | Descriptive,  Cross-sectional. *Clinical, 24-Hour Recall, Anthropometric, Questionnaire, Accelerometer* | Inservice*,  Elementary | - Dietary intake - Dietary pattern analysis - Body mass index |  | - Physical activity - Smoking status - Fasting bloods |
| Hasan 2019, Indonesia^14^ | To assess the knowledge attitudes and practices about balanced nutrition guidelines and metabolic syndrome in high school teacher with central obese. | Descriptive,  Cross-sectional. *Questionnaire, Interviews, Clinical, Anthropometric* | Inservice,  Secondary, High school | - Knowledge, attitudes, and practices about balanced nutrition - Body mass index - Waist circumference |  | - Fasting bloods |
| Hemati 2020, Iran^15^ | To investigate the effect of an educational intervention on the nutrition literacy of primary school teachers in Yasuj. | Intervention, Quasi-experimental. *Questionnaire*  R | Inservice,  Primary | - Nutrition literacy |  |  |
| Husain 2021, Kuwait^16^ | To evaluate the level of general nutrition knowledge and demographic variations in knowledge in a sample of students attending the College of Basic Education in Kuwait. | Descriptive,  Cross-sectional. *Questionnaire, Anthropometric* | Pre-service*,  Kindergarten, Primary, Intermediate | - Nutrition knowledge - Cooking and food acquisition responsibilities - Sourcing nutrition information - Body mass index |  |  |
| Jose Rombaldi 2012, Brazil^17^ | To evaluate physical education teachers’ knowledge about the associations between four behavioural factors (sedentary lifestyle, smoking, abusive alcohol intake, and inadequate eating) and eight diseases (diabetes, hypertension, AIDS, osteoporosis, lung cancer, depression, liver cirrhosis and acute myocardial infarction). | Descriptive,  Cross-sectional. *Questionnaire, Anthropometric* | Inservice,  Primary | - Body mass index - Nutritional characteristics | - Teaching characteristics | - Physical activity - Smoking status - Health - Chronic disease knowledge and impact of risk factors - Demographic - Socio-economic-status |
| Katsagoni 2019, Greece^18^ | To explore teachers' nutrition knowledge, beliefs, and attitudes and to examine the effectiveness of an electronic learning (e-learning) program in teachers nutrition knowledge. | Intervention, Experimental Cross-sectional. *Questionnaire*  R | Inservice,  Primary, Secondary | - Nutrition knowledge - Teacher nutrition attitudes and beliefs *(also contains professional-related content in this construct)* | - Barriers to nutrition education delivery |  |
| LeCheminant 2015, United States of America^19^ | To examine the individual associations of selected health behaviours (exercise, fruit, vegetable intake, restful sleep, smoking and alcohol consumption) with work-related outcomes among employees of a school district as well as the extent that multiple behaviours are related to these outcomes. | Descriptive,  Cross-sectional. *Questionnaire, Anthropometric* | Inservice*,  High school, Junior high, Elementary | - Dietary intake |  | - Exercise practices - Restful sleep - Cigarette smoking - Self-reported health - Productivity (job performance, absenteeism) - Satisfaction (with job and life) - Emotional health (depression, stress, loneliness) - Alcohol consumption |
| LeCheminant 2017, United States of America^20^ | To examine the health behaviour changes and mental-health and job-related outcomes of school-based employees over two years while participating in a worksite wellness program. | Intervention, Non-experimental design (no control group). *Questionnaire* | Inservice*,  High school, Junior high, Elementary | - Dietary intake |  | - Exercise - Mental health related outcomes (stress, depressions, life satisfaction, loneliness score) - Job related outcomes (job performance, absenteeism, job-related satisfaction) - Self-rated health; Restful sleep - Smoking status and intake - Alcohol consumption |
| Lemon 2013, United States of America^21^ | To describe the development of measures of worksite descriptive social norms for weight loss, physical activity, and eating behaviours. | Measurement, Cross-sectional. *Questionnaire, Anthropometric*  VR | Inservice*, High school | - Worksite related social norms and social support for weight loss - Eating behaviour inventory - Currently trying to lose weight |  | - Perceived organizational commitment to employee health - Physical activity - Worksite related social norms and social support for physical activity |
| Melville 2006, United States of America^22^ | To capture the wellness practices of pre-service physical educators, teaching interests, about subject area levels and grade preferences. | Descriptive,  Cross-sectional.  *Questionnaire, Anthropometric* | Pre-service,  Middle school, Secondary, Elementary | - Body mass index - Wellness practices |  | - Physical activity - Teaching Interests |
| Merrill 2014, United States of America^23^ | To evaluate the effectiveness of a worksite wellness program in decreasing health related risk. | Intervention, Pre-post/Non-Randomised-experimental. *Questionnaire, Clinical, Anthropometric* | Inservice*,  Elementary, High school, Junior high | - Dietary intake |  | - Physical activity - Health status - Life satisfaction - Sleep quality - Smoking status - Seat belt use |
| Monica 2018, India^24^ | To determine risk of obesity among female schoolteachers along with various factors and health problems associated with it. | Descriptive,  Cross-sectional. *Clinical, Questionnaire, Interviews, Physical Assessment, Anthropometric* | Inservice,  Primary, Middle school, High school, Higher secondary | - Dietary intake - Body mass index |  | - Physical activity - Health problems - Blood pressure |
| Motamedrezaei 2013, Iran^25^ | To survey the influence of nutrition and nutritional health education on the awareness of female elementary school teachers. | Descriptive,  Quasi.  *Questionnaire*  V | Inservice,  Elementary | - Nutrition knowledge - Food hygiene |  |  |
| Mullaney 2008, Ireland^26^ | To examine the relationship between nutrition education and lifestyle behaviours in a select group of third level students. | Descriptive,  Longitudinal.  *Questionnaire, Anthropometric* | Pre-service,  Secondary | - Body mass index - Nutrition and related lifestyle knowledge - Dietary intake - Attitudes to healthy eating and lifestyle |  | - Physical activity |
| O'Dea 2001, Australia^27^ | To examine the knowledge, beliefs, and attitudes about weight control and eating disorders among trainee home economics and physical education teachers and to assess their body image and weight control practices. | Descriptive,  Cross-sectional. *Questionnaire, Anthropometric*  V | Pre-service,  Secondary | - Food habits - Body image - Weight control practices - History of eating disorders - Knowledge, behaviour and attitudes about weight control and eating disorders - Body mass index | - Knowledge, attitudes, behaviours about suitable weight control advice for students | - Physical self-esteem |
| Rafiroiu 2005, United States of America^28^ | To assess knowledge, attitudes, body size perceptions and weight control behaviours of a sample of public-school teachers from elementary, middle, and high schools in a Southern state. | Descriptive,  Descriptive. *Questionnaire, Anthropometric* | Inservice*,  Elementary, Middle school, High school, Kindergarten | - Nutrition knowledge - Nutrition attitudes - Weight control behaviours - Body size perception - Body mass index | - Perceived role of school cafeteria | - Sources of Nutrition information |
| Russell-Mayhew 2012, Canada^29^ | A pre-post mixed methods design was used to assess the influence of the interactive professional development training on pre-service teacher’s values concerning body image, size acceptance, and eating attitudes and behaviours (teachers as being). | Intervention, Pre-post. *Questionnaire, Focus Groups, Anthropometric* | Pre-service,  Secondary | - Body mass index - Weight related issues - Disordered eating - Body satisfaction and acceptance of body shapes |  | - Self-efficacy to address weight related issues in their teaching practices |
| Schultz 2020, United States of America^30^ | To examine the effects of physical activity, diet quality and stress on cardiometabolic risk factors among school employees and explore the interaction between stress and health behaviours. | Descriptive,  Cross-sectional. *Clinical, Questionnaire, Anthropometric* | Inservice*,  Elementary | - Body mass index - Dietary intake - Dietary index(s) calculated |  | - Physical activity - Job strain score - Perceived occupational stress (Psychological demands score and decision latitude score) - Sitting time in hours - Blood sample |
| Thaha et al, 2021, Indonesia^31^ | Aims to measure changes in autonomy in groups that have been given nutrition education by applying the self-determination theory concept. | Intervention, Pre-post. *Questionnaire* | Inservice,  Senior school | - Self-regulation of diet |  |  |
| Vio 2018, Chile^32^ | Through focus groups, we explored 22 third- to fifth-grade teachers' perceptions about their eating habits, including barriers and facilitators to healthy eating. It also explored teachers' thoughts about how to teach students healthy eating habits. | Descriptive,  Qualitative. *Focus Groups* | Inservice,  Primary | - Teachers’ eating habits - Culinary habits |  | - Information and Communication Technologies and participative activities |
| Wang 2016, China^33^ | To examine the effectiveness of a holistic school-based nutrition programme using the health-promoting school approach, on teachers' knowledge, attitudes, and behaviour in relation to nutrition in rural China. | Intervention, Cluster-randomised intervention trial. *Questionnaire* | Inservice,  Middle school | - Dietary intake - Nutrition knowledge - Nutrition attitudes |  |  |
| Wilkinson 2013, United States of America^34^ | To assess differences in self-regulation of attitudes towards engaging in exercise and eating a healthy diet between physical education teacher education students and general education students, and between male students and female students. | Descriptive,  Cross-sectional. *Questionnaire* | Pre-service, Kindergarten to grade 12 | - Self-regulation of diet |  | - Self-regulation of exercise |
| Woynarowska-Soldan 2015, Poland^35^ | To present the concept, methods of implementation, results, and experiences from the first stage of the three-year project on school staff health promotion carried out within the framework of the health-promoting school network in Poland. | Intervention. *Questionnaire* | Inservice*,  Primary, Lower secondary | - Frequency of practising the five groups of health enhancing behaviour: nutrition, physical activity, sleep, rest, and behaviour relating to mental health, safety, taking care of one’s body and avoiding risky behaviour |  |  |
| Yager 2017, Australia^36^ | To compare the body image, eating, and exercise attitudes and behaviours of first-year education university students who were specialising in health and physical education, with those who were not specialising in this area. | Descriptive,  Cross-sectional. *Questionnaire, Anthropometric* | Pre-service,  Primary, Secondary | - Body mass index - Weight change behaviours (includes Disordered eating) - Body image/dissatisfaction - Body image/drive for thinness - Body image/drive for muscularity |  | - Excessive exercise |
| Yager 2009, Australia^37^ | To investigate and compare body image, body dissatisfaction, dieting, disordered eating, exercise and eating disorders among trainee health education/physical education and non- health education/physical education teachers. | Descriptive,  Cross-sectional. *Questionnaire, Anthropometric* | Pre-service,  Primary, Secondary | - Body mass index - Disordered eating - Dieting behaviours - Body weight perceptions - The figure rating scale - Past and current treatment of eating disorder |  | - Excessive exercise |

Legend: *Included non-teacher participants, ^Direct excerpt from source paper, V Description of validity testing provided, R Description of reliability testing provided.

**Table 2** Teacher Professional FN Focused Primary Study Aim

| **Source (Author, Year, Country)** | **Primary Study Aim^**  **Teacher Professional FN** | **Study [Type, Design] and** *Data collection method(s)* | **Teacher Participants (Service Status, Level taught)** | **Personal Food and Nutrition Factors Explored** | **Professional Food and Nutrition Factors Explored** | **Other Health and Wellbeing Factors Explored** |
| --- | --- | --- | --- | --- | --- | --- |
| Arcan 2013, United States of America^38^ | To examine frequencies of classroom and school food practices and beliefs of kindergarten and first grade teachers in schools located on American Indian reservation. | Descriptive, Prospective.  *Questionnaire*  V | Inservice*,  Elementary | - Eating habits | - Classroom food practices - School-wide food practices - Beliefs regarding the school-food environment - Food-related school policy |  |
| Beffa-Negrini 2007, United States of America^39^ | Design, implement and evaluate a web-based program in food safety for secondary science teachers. | Intervention, Pre-post.  *Questionnaire*  V | Inservice,  Primary, Junior high, Secondary | - Food safety skills, knowledge, and practices - Food safety behaviours | - Food safety teaching self-efficacy and confidence - Intention to teach food safety in coming year | - Sourcing food safety information |
| Chrisman 2020, United States of America^40^ | To examine (1) the prevalence and strategies of using nutrition lessons and MyPlate guidelines among kindergarten-12 teachers and principals in schools and classrooms, and how the use may differ by school level and demographic and teaching characteristics; (2) barriers to and facilitators of using MyPlate; and (3) how teachers might use MyPlate and their suggested strategies to improve its use. | Descriptive,  Cross-sectional.  *Questionnaire, Anthropometric*  V | Inservice,  Elementary, Middle school, High school | - Dietary intake - Body mass index | - Use of nutrition resources particularly MyPlate | - Physical activity - Sleep - Sedentary behaviour |
| Coccia 2020, United States of America^41^ | To determine the impact of personal health, body mass index, nutrition knowledge and self-efficacy on classroom food-related beliefs and practices | Descriptive,  Cross-sectional.  *Questionnaire, Anthropometric*  V | Pre-service,  Elementary | - Personal health index - Body mass index | - Nutrition teaching self-efficacy - Beliefs and attitudes about the school food environment - Classroom food practices - Nutrition knowledge focused on children and teaching |  |
| Costello 2005, United States of America^42^ | To develop a web-based food safety course for high school teachers that teach food and culinary practices and evaluate its effectiveness on knowledge gained. | Intervention,  Pre-post.  *Questionnaire* | Inservice,  High school | - Food safety |  | - Effectiveness of the course |
| Eley 2021, England, France, Hungary, Portugal^43^ | To explore school educators’ attitudes,  behaviours and knowledge towards food hygiene, safety, and education. | Descriptive, Qualitative.  *Interviews, Focus groups* | Inservice,  Secondary | - Food safety | - Food safety teaching self-efficacy - Resources used to teach food safety |  |
| Elorinne 2020, Finland^44^ | Examines Finnish home economic teachers attitudes towards food waste and sustainable food education, and how these attitudes align with the teachers personal and pedagogical practice. | Descriptive,  Cross-sectional. *Questionnaire* | Inservice,  Secondary, Elementary | - Food waste attitudes and practices - Food value orientations | - Food waste pedagogical practices |  |
| Endres 2001, United States of America^45^ | To pilot a touch-screen kiosk to assess basic food safety knowledge and provide immediate feedback on food safety principles to high school science teachers (potential food safety teachers of young persons) and students. | Intervention, Cross sectional  *Questionnaire* | Inservice,  High school | - Food safety |  |  |
| Falkenbach 2018, Brazil^46^ | To develop a questionnaire and assess knowledge, attitudes, and practices of early years schoolteachers. | Description, Cross sectional descriptive. *Questionnaire* | Inservice,  Primary, Elementary | - Knowledge about nutrition and food - Personal health perceptions | - Attitudes to school food and nutrition environment - Teacher school food and nutrition practices |  |
| Findholt 2016, United States of America^47^ | To examine the classroom food practices, personal eating behaviours at school, beliefs about the school food environment, and nutrition knowledge of rural elementary and middle school teachers. | Descriptive,  Cross-sectional.  *Questionnaire*  V | Inservice, Elementary | - Nutrition knowledge - Eating behaviours at school | - Classroom food practices - Beliefs about the school food environment - Nutrition knowledge regarding student requirements |  |
| Hamilton 2021, United States of America^48^ | Examined associations between teacher demographic and individual factors and their food-related practices and modelling in the classroom. | Description,  Cross-sectional. *Questionnaire,*  *Anthropometric* | Inservice,  Elementary, Middle school, High school | - Nutrition knowledge - Dieting status - Body mass index - Personal health index | - Classroom food practices |  |
| Jones 2015, United State of America^49^ | To determine barriers to nutrition education, nutrition education resources used, and the relationship between nutrition knowledge and whether public school teachers in California teach nutrition in the classroom. | Description,  Cross-sectional.  *Questionnaire*  VR | Inservice,  PreK-12 | - Nutrition knowledge | - Barriers to nutrition education teaching - Resources used by teachers to plan nutrition lessons |  |
| Kaschalk-Woods 2021, United Statess of America^50^ | To examine the effects of training and implementation of Forecasting Your Future: Nutrition Matters on teacher’s self-efficacy to teach nutrition and teachers nutrition knowledge. | Intervention, Pre-post experimental. *Questionnaire, Interviews* | Inservice,  High school | - Nutrition knowledge | - Nutrition teaching self-efficacy |  |
| Kinsler 2012, United States of America^51^ | The purpose of this study was to assess nutrition-related knowledge and self-efficacy of teachers from a large urban school district in Los Angeles County. | Descriptive, Pilot descriptive. *Questionnaire* | Inservice,  Elementary | - Nutrition knowledge | - Nutrition teaching self-efficacy - Nutrition knowledge, student nutrition and dietary intake requirements |  |
| Kubik 2002, United States of America^52^ | Examined classroom food practices and eating behaviour of middle school teachers from 16 schools in a metropolitan area, located in the upper Midwest. | Descriptive, Cross-sectional.  *Questionnaire*  V | Inservice,  Middle school | - Teacher eating patterns at school - Dietary intake - Personal health index | - Classroom food practices - School food environment, eating and teens |  |
| Laguna 2020, United States of America^53^ | To add to literature by investigating elementary school teacher’s beverage intake at school and how such consumption relates to that of their students. | Descriptive,  Cross-sectional. *Questionnaire* | Inservice, Elementary | - Beverage consumption at school | - Role modelling of beverage consumption in front of students and in the classroom - Perceptions on ways to improve water consumption in students |  |
| Metos 2019, United States of America^54^ | Examined teacher views and practices regarding nutrition education. | Descriptive,  Cross-sectional.  *Questionnaire*  VR | Inservice,  Elementary | - Personal health index | - Nutrition teaching self-efficacy - Classroom nutrition practices - Nutrition attitudes and beliefs |  |
| Molloy 2008, Ireland^55^ | To explore teachers’ knowledge about water and the perceived barriers to allowing children access to water during lesson time. | Descriptive,  Qualitative. *Interviews* | Inservice,  Primary | - Knowledge on drinks, water, and hydration - Fluid recommendations for adults (and children) | - Opinions and practices of water in classroom |  |
| Oldewage-Theron 2012, South Africa^56^ | The main objective of this pilot study was to determine the impact of a nutrition education training programme on the nutrition knowledge of Life Orientation educators in public schools in South Africa. | Intervention, Non-randomised experimental. *Questionnaire* | Inservice*,  Primary | - Self-rated perceptions of nutrition knowledge | - Nutrition education practices and resources used or needed. - Nutrition education topics to be included in the syllabus |  |
| Parker 2020, United States of America^57^ | To examine associations between teachers' diet quality and their nutrition-related classroom practices (e.g., rewarding students with food, modelling healthy diet behaviours). | Descriptive,  Cross-sectional.  *Questionnaire, Anthropometric* | Inservice*,  Elementary, Middle school | - Dietary intake - Body mass index | - Nutrition-related classroom practices |  |
| Perikkou 2015, Cyprus^58^ | Teachers’ attitudes about school food environments and their readiness to implement school-based nutrition programs were investigated. | Descriptive,  Epidemiological study.  *Questionnaire* | Inservice,  Elementary, Primary | - Dietary intake - Personal health index | - Attitudes and barriers to implementing health education program - Teacher readiness to take and accomplish |  |
| PflughPrescott 2018, United States of America^59^ | Examined the relationships between teacher self-reported body mass index, eating competence status and scores, cooking attitudes, and cooking behaviours with their attitudes toward tasting lessons implemented as a part of Fuel for Fun, a multicomponent, school-based intervention. | Intervention,  Cross-sectional.  *Questionnaire, Anthropometric* | Inservice,  Elementary, Primary | - Eating competence - Frequency of home meal preparation - Average time spent preparing a meal - Cooking attitudes - No' of college nutrition courses taken - Body mass index | - Teacher experiences with Fuel for Fun lessons - Rating of the lesson delivery of Fuel for Fun educators | - Physical activity - Whether they consider themselves physically active |
| Pivarnik 2009, United States of America^60^ | To implement a survey to assess high school and transition special education teachers in Rhode Island, Connecticut, and Massachusetts for food safety knowledge and attitudes toward the use of food safety education for special needs students. | Descriptive,  Cross-sectional.  *Questionnaire*  VR | Inservice*,  High school | - Food safety knowledge | - Food safety attitudes - Curriculum audit - Food preparation practices in the classroom |  |
| Ronto 2016, Australia^61^ | Examined home economics teachers' perspectives of the importance, curriculum, self-efficacy, and food environments regarding food literacy in secondary schools in Australia. | Descriptive,  Cross-sectional.  *Questionnaire*  V | Inservice,  Secondary | - Self-efficacy and attitudes towards food literacy | - School food environment - Importance of aspects of food literacy |  |
| Rossiter 2007, Canada^62^ | The knowledge, attitudes and eating behaviours of prospective teachers as determinants of intended classroom food practices and the school environment and its potential impact on classroom food practices were examined and explored. | Descriptive,  Cross-sectional.  *Questionnaire*  V | Pre-service,  Elementary, Secondary | - Nutrition knowledge index - Prospective teacher eating patterns at school - Dietary intake - Personal health index | - Intended classroom practices - School food environment index |  |
| Russell-Mayhew 2015, Canada^63^ | The aim of the current study was to assess the impact of a three-hour professional development workshop delivered to preservice teachers who will be responsible for teaching health education (i.e., elementary specialists). | Intervention, Pre-post.  *Questionnaire, Anthropometric* | Pre-service,  Elementary | - Eating attitudes - Anti-fat attitudes - Body satisfaction - Body mass index | - Self-efficacy in participating in weight and body image related contexts within the school environment |  |
| Selvam 2017, India^64^ | To assess effect of a short training programme on non-communicable diseases, particularly diabetes on schoolteachers and on students who were in turn educated by them. Lifestyle changes made by both groups were assessed six months later. | Intervention, Pre-post.  *Questionnaire* | Inservice,  High school | - Improvement in lifestyle practices (including healthy eating, physical activity, yoga, and meditation) |  | - Information shared with colleagues (including healthy food habits, physical activity, awareness of diabetes, diabetes complications) |
| Snelling 2012, United States of America^65^ | To assess the health status of teachers in a large urban school district and understand their beliefs and self-efficacy on the intersection of learning and health for the students they teach. | Descriptive,  Exploratory. *Questionnaire, Anthropometric* | Inservice,  Elementary, Middle school, High school | - Nutrition patterns - Body mass index | - Teachers’ beliefs and self-efficacy on school health education, as well as teachers perceived role in teaching health | - Physical activity - Personal Health |
| Thompson 2007, United States of America ^66^ | To use a validated instrument to determine the attitudes and knowledge of high school teachers regarding food irradiation, and to determine the correlations among their knowledge and attitudes and certain demographic variables. | Descriptive,  Cross-sectional. *Questionnaire*  VR | Inservice,  High school | - Food attitudes |  |  |
| Wai-ling 2004, China^67^ | This study was conducted to reflect critically on implications of the issue on the health and well-being of young people in Hong Kong. | Descriptive,  Cross-sectional. *Questionnaire, Interviews* | Inservice,  Secondary | - Food advertisement knowledge and behaviour |  |  |

Legend: *Included non-teacher participants, ^Direct excerpt from source paper, V Description of validity testing provided, R Description of reliability testing provided.

**Table 3** Student FN Focused Primary Study Aim

| **Source (Author, Year, Country)** | **Primary Study Aim^**  **Student FN** | **Study (Design used, Data type collected)** | **Teachers (Type, Level taught)** | **Personal** | **Professional** | **Other Health and Wellbeing Factors explored** |
| --- | --- | --- | --- | --- | --- | --- |
| Al-Refaee et al., 2013, Kuwait^68^ | To study nutrition knowledge, attitude, and practice (including free time activities) of adolescent schoolgirls in Kuwait. To develop a strategy for improving their health and nutritional status. | Descriptive,  Prospective. *Questionnaire, Anthropometric* | Inservice,  High school | - Primary sources of nutrition information - Body mass index - Food habits and eating behaviour - Knowledge of nutrients score - Knowledge of nutrients function score - Nutrition attitudes - Nutrition practice |  | - Physical activity - Lifestyle score |
| Antwi 2020, Ghana^69^ | To evaluate the effect of a six-week nutrition education intervention on the nutrition knowledge, attitude, practices, and nutrition status of school-age children (aged six- twelve years) in basic schools in Ghana. | Intervention,  Randomised control trial/pre-post. *Questionnaire* | Inservice,  Primary | - Nutrition knowledge - Nutrition attitudes and practices |  |  |
| Hyska 2020, Albania^70^ | To assess the nutritional status and the nutrition-related knowledge, attitudes, and practices of Albanian school-aged children. | Descriptive,  Cross-sectional. *Questionnaire/Interview* | Inservice,  Primary, Secondary | - Food and nutrition knowledge and perceptions | - The nutritional environment and related context in their respective schools - Nutrition knowledge including child nutrition and health |  |
| Lopez-Barron 2015, Mexico^71^ | To assess whether there is an association between food availability in children’s environments and their food consumption with body mass index, z-score, and waist circumference. | Descriptive,  Cross-sectional. *Questionnaire*  R | Inservice,  Elementary | - Dietary intake, Food composition (school food environment and what foods were available in this space) |  | - Physical activity |
| Linnell 2016, United States of America^72^ | To examine whether teacher characteristics and implementation factors influenced the effectiveness of a multicomponent nutrition education program to improve nutrition-related knowledge and behaviour among fourth-grade children. | Intervention,  Pre-post.  *Questionnaire, Observations* | Inservice,  Primary, Elementary | - Knowledge about nutrition | - Nutrition teaching self-efficacy - Teachers self-reported degree of completion of the program |  |
| Lozada 2008, Mexico^73^ | To establish the school eating habits of Mexican children, who are prone to obesity and later to high rates of adult chronic diseases. | Descriptive,  Cross-sectional. *Questionnaire, Interviews* | Inservice,  Primary, Secondary, High school | - Teacher school lunches | - Food policy at school |  |
| Machado 2019, United States of America^74^ | This manuscript describes the design and rationale for the multi-pronged Intervention to Increase secondary student participation in school lunch study, as well as participant baseline characteristics | Intervention,  Quasi.  *Questionnaire* | Inservice,  Middle school, High school | - School lunch participation - Attitudes, and perception of school lunch | - Modelling and encouraging of healthy eating behaviours | - General health |
| McVey 2007, Canada^75^ | To examine the influence of the comprehensive, universal-selective intervention in improving body satisfaction and size acceptance and in reducing the internalization of media ideals, weight-based teasing, disordered eating and weight-loss or muscle-gaining behaviours. | Intervention,  Pre-post.  *Questionnaire, Anthropometric* | Inservice,  Middle school | - Body mass index Disordered eating - Body satisfaction - Internalisation of media stereotypes | - Perception of the school climate |  |
| Okeyo 2020, South Africa^76^ | To evaluate the food and nutrition environment in terms of government policy programs, nutrition education provided, and foods sold at secondary schools in the Eastern Cape province to assess how healthy this environment is for learners. | Descriptive,  Multi-stage cluster sampling method. *Interview* | Inservice,  Secondary | - Training in nutrition and knowledge of the food based dietary guideline’s |  |  |
| Shah 2010, India^77^ | To evaluate the impact of a school-based health and nutritional education programme on knowledge and behaviour of urban Asian Indian school children. | Intervention,  Non-randomised experimental *Questionnaire* | Inservice, Senior secondary | - Nutrition knowledge and behaviours |  |  |
| Talip 2017, Brunei^78^ | To explore the perceptions, practices, and attitudes towards healthy eating in Bruneian primary school children. | Descriptive,  Qualitative.  *Focus Groups* | Inservice,  Primary | - Perceptions of Healthy Eating | - Perceptions of what healthy eating should be for students or should look like |  |

Legend: *Included non-teacher participants, ^Direct excerpt from source paper, V Description of validity testing provided, R Description of reliability testing provided.

**Table 4** Student and Teacher Personal FN Focused Primary Study Aim

| **Source (Author, Year, Country)** | **Primary Study Aim^**  **Student/ Teacher Personal FN** | **Study (Type, Design used, Data type collected)** | **Teachers (Type, Level taught)** | **Personal Food and Nutrition Factors Explored** | **Professional Food and Nutrition Factors Explored** | **Other Health and Wellbeing Factors Explored** |
| --- | --- | --- | --- | --- | --- | --- |
| Berger-Jenkins 2014, United States of America^79^ | To evaluate whether a comprehensive obesity prevention program that targets children and school staff in an under severed Hispanic community affects obesity related knowledge, attitudes, and behaviours among both students and staff. | Intervention, Longitudinal. *Questionnaire* | Inservice*,  Elementary | - Nutrition knowledge - Nutrition attitudes - Behaviours |  | - Physical activity behaviour - Physical activity - Knowledge - Self-efficacy to lead a physical activity lifestyle - Readiness to change their physical activity |
| Gaglianone 2006, Brazil^80^ | To analyse the development and implementation of the RRIDA Project Nutrition Education component and its impact on knowledge and attitudes regarding healthy eating habits of both students and teachers. | Intervention, Randomised control trial. *Questionnaire, Interviews* | Inservice,  Primary | - Nutrition knowledge | - Teaching attitudes and modelling |  |
| Kupolati 2019, South Africa^81^ | Implemented a context-specific nutrition education program developed for Grade four-seven teachers and tested the hypothesis that the nutrition education program would significantly improve the nutrition knowledge, attitudes, and dietary practices of the teachers and the learners in the treatment school. | Intervention, Quasi experimental. *Questionnaire*  R | Inservice,  Primary | - Knowledge - Attitudes - Practices/Dietary practices | - Nutrition knowledge: current dietary recommendations for children |  |
| Laurie 2017, South Africa^82^ | To assess knowledge, perceptions, and practices on food production amongst learners and educators, gardening activities and management of school food gardens in schools participating in the National School Nutrition Programme. | Descriptive,  Cross-sectional. *Questionnaire* | Inservice,  Primary | - Attitudes towards eating fruits and vegetables | - Integration of food gardening into the curriculum | - The value of a school food garden and its role in nutrition - Sources of gardening information |
| Lee 2016, Korea^83^ | Investigated the effect of switching normal diet to vegetarian diet rich in vegetables and fruits for school food service and home meal on the nutritional status, bowel habit improvement and stress reduction of teachers and adolescents. | Intervention, Pre-post. *Questionnaire, Anthropometric, Clinical* | Inservice,  Middle school, Junior high | - Vegetarian diet - Eating behaviour - Body mass index - Dietary habits/eating behaviours |  | - Bowel health - Perceived stress - Fasting bloods |
| O'Dea 2016, Australia^84^ | To examine change in nutrition and physical activity knowledge, self-efficacy, and attitudes in a cohort of 23 teachers and 304 year five and six children after the "Healthy Active Kids" online program. | Intervention, Pre-post. *Questionnaire* | Inservice,  Primary | - Nutrition knowledge - Attitudes towards diet and nutrition | - Confidence in teaching about diet and nutrition in the classroom | - Attitudes towards Physical activity - Frequency of physical activity |

Legend: *Included non-teacher participants, ^Direct excerpt from source paper, V Description of validity testing provided, R Description of reliability testing provided.

**Table 5** Other Focused Primary Study Aim

| **Source (Author, Year, Country)** | **Primary Study Aim^**  **Other** | **Study (Type, Design used, Data type collected and data collection methods)** | **Teachers (Type, Level taught)** | **Personal Food and Nutrition Factors Explored** | **Professional Food and Nutrition Factors Explored** | **Other Health and Wellbeing Factors Explored** |
| --- | --- | --- | --- | --- | --- | --- |
| Barwood 2020, Australia^85^ | Investigate the use of transformational games to engage young people in healthier food choices, game player knowledge of nutrition and the design and evaluation of a transformational game as a pedagogical device for nutrition education. | Intervention, Randomised control trial. *Questionnaire* | Pre-service*,  Secondary | - Digestion knowledge score - Body food use score - Healthy food choices score - Attitudes to food and drinks score - Future food intentions |  | - Post-trial game attitude score |
| Canchola 2015, United States of America^86^ | Investigated whether dietary patterns are associated with endometrial cancer risk among women in the California Teachers Study cohort. | Descriptive,  Cohort. *Questionnaire, Anthropometric, Linkage data* | Inservice*,  Elementary, High school, Junior high | - Dietary intake - Dietary pattern analysis - Body mass index |  | - Medical history - Health - Lifestyle and behaviours - Cancer diagnosis - Hormone therapy - Menopause status - Physical activity - Smoking status - Alcohol intake |
| Chang 2007, United States of America^87^ | Investigated the role of isoflavones and isothiocyanates, as well as other dietary factors, in the development of ovarian cancer in the prospective California Teachers Study cohort. | Descriptive,  Cohort. *Questionnaire, Anthropometric, Linkage Data* | Inservice*,  Elementary, High school, Junior high | - Dietary intake - Body mass index |  | - Physical activity - Smoking status - Alcohol intake - Supplement use |
| Chang 2008, United States of America^88^ | To investigate the association between dietary patterns and risk of ovarian cancer in the prospective California Teachers Study cohort. | Descriptive,  Cohort. *Questionnaire, Anthropometric, Linkage Data* | Inservice*,  Elementary, High school, Junior high | - Dietary intake - Dietary pattern analysis - Body mass index |  | - Physical activity - Smoking status - Alcohol intake |
| Eng 2018, Malaysia^89^ | To derive dietary patterns empirically and to examine the consistency and generalizability of patterns across sex, ethnicity, and urban status in a working population. | Descriptive,  Cohort. *Questionnaire, Anthropometric* | Inservice,  Primary, Secondary | - Dietary intake - Dietary pattern analysis - Body mass index |  | - Physical activity - Family and medical history - Smoking status |
| Eng 2020, Malaysia^90^ | To examine the association between dietary patterns and overweight obesity and to explore their dose response association using a restricted cubic spline mode. | Descriptive,  Cohort. *Questionnaire, Anthropometric* | Inservice,  Primary, Secondary | - Dietary intake - Body mass index - Dietary Pattern Analysis |  | - Physical activity - Smoking |
| Esmaillzadeh 2006, Iran^91^ | To assess the association of fruit and vegetable intakes with blood C-reactive protein concentrations and the prevalence of the metabolic syndrome among female teachers aged 40-60 years living in Tehran | Descriptive,  Cross-sectional. *Clinical, Questionnaire, Anthropometric*  V | Inservice,  Primary, High school | - Dietary intake - Body mass index |  | - Physical activity - Blood pressure, Fasting bloods - Smoking habits - Menopause status - Medical history - Current use of medications |
| Esmaillzadeh 2007, Iran^92^ | To evaluate the association of major dietary patterns characterized by factor analysis with insulin resistance and the metabolic syndrome among women. | Descriptive,  Cross-sectional. *Clinical, Questionnaire, Anthropometric*  V | Inservice,  Primary, High school | - Dietary intake - Dietary pattern analysis - Body mass index |  | - Physical activity - Smoking habits - Medical history - Current use of medications - Menopause status - Blood pressure, Fasting Bloods |
| Esmaillzadeh 2012, Iran^93^ | Aimed to assess legume intake in relation to adhesion molecules and inflammatory biomarkers among Iranian women. | Descriptive,  Cross-sectional. *Clinical, Questionnaire, Anthropometric*  V | Inservice,  Primary, High school | - Dietary intake - Dietary pattern analysis - Body mass index |  | - Physical activity - Smoking - Use of medications - Stroke and medical history - Family history of diabetes - Fasting bloods |
| Haridass 2018, United States of America^94^ | Examine the association between diet quality indexes and pre-and postmenopausal breast cancer risk in a large prospective cohort. | Descriptive,  Cohort. *Questionnaire, Linkage Data; Anthropometric* | Inservice*,  Elementary, High school, Junior high | - Dietary intake - Body mass index - Dietary index(es) calculated |  | - Physical activity - Family history of breast cancer and Chronic Disease - Smoking status - Age of menarche and menopause status - Alcohol intake |
| Horn-Ross 2002, United States of America^95^ | To address the relationship between recent adult diet and breast cancer risk during the first two years of follow up of the large California Teachers Study cohort. | Descriptive,  Cohort. *Questionnaire, Linkage Data, Anthropometric* | Inservice*,  Elementary, High school, Junior high | - Dietary intake - Body mass index |  | - Physical activity - Menstrual and reproductive events - Use of exogenous estrogen - Medication - Personal and family history of cancer and Chronic Disease - Screening behaviours - Smoking status/Tobacco use - Indications of exposure to potential environmental hazards - Alcohol intake - Vitamin Use |
| Jafar 2020, Indonesia^96^ | To examine the effects of school-based nutrition using the self-determination theory on nutrition knowledge, attitudes, and practices of the senior secondary schoolteacher with metabolic syndrome risk. | Intervention, Pre-post. *Clinical, Questionnaire, Anthropometric* | Inservice,  Senior school | - Nutrition knowledge, attitudes, and practices - Body mass index - Waist circumference |  | - Blood pressure, Fasting bloods - Metabolic Syndrome Classification |
| Khatibi 2019, Iran^97^ | To examine the relationship between dietary patterns and inflammatory markers including serum high sensitivity C-reactive protein and interleukin 17A in females. | Descriptive,  Cross-sectional. *Questionnaire, Anthropometric, Clinical* | Inservice*,  Elementary, High school | - Dietary intake - Dietary pattern analysis - Body mass index |  | - Physical activity - Fasting bloods, Blood pressure - Vitamin Use |
| Link 2013, United States of America^98^ | To evaluate dietary patterns and their relation to breast cancer risk in a large cohort of women. | Descriptive,  Cohort.  *Questionnaire, Linkage Data, Anthropometric* | Inservice*,  Elementary, High school, Junior high | - Dietary intake - Dietary pattern analysis |  | - Use of exogenous estrogen - Use of vitamins - Potential breast cancer risk factor - Personal lifestyle characteristics - Menstrual and reproductive events - Medications - Personal and family history of cancer - Physical activity - Consumption of alcohol - Personal and family history of chronic disease - Tobacco Use |
| Pacheco 2020, United States of America^99^ | Examined the prospective association of baseline sugar sweetened beverages consumption with incident cardiovascular disease in 106 178 women free from cardiovascular disease and diabetes mellitus in the California Teachers Study, a cohort of female teachers and administrators, followed since 1995. | Descriptive,  Cohort. *Questionnaire, Linkage Data, Anthropometric*  V | Inservice*,  Elementary, High school, Junior High | - Dietary intake - Dietary pattern analysis - Body mass index |  | - Cardio-vascular disease status and assessment - Alcohol intake - Smoking Status - Physical activity |
| Rana 2010, Australia^100^ | To assess the effectiveness of using a Health Promoting Schools framework to deliver a nutrition intervention in schools. | Intervention, Non-randomised experimental study. *Questionnaire, Interviews* | Inservice*,  Primary, Secondary | - Increases in healthy food choices |  |  |
| Reynolds 2004, United States of America^101^ | To describe characteristics associated with active and passive smoking in a large cohort of women to identify possible confounders of the relationship between smoking exposures and breast cancer risk. | Descriptive,  Cohort. *Questionnaire, Linkage Data, Anthropometric* | Inservice*,  Elementary, High school, Junior high | - Dietary intake - Body mass index |  | - Smoking status - Physical activity - Alcohol intake - Supplement Use |
| Sakhaei 2018, Iran^102^ | To investigate the association between Dietary Approaches to Stop Hypertension and the Mediterranean dietary patterns with circulating C-reactive protein and interleukin -17A levels. | Descriptive,  Cross-sectional. *Clinical, Questionnaire, Anthropometric* | Inservice*,  Elementary, High school | - Dietary intake - Dietary pattern analysis - Dietary index(es) calculated - Body mass index |  | - Physical activity - Smoking status - Chronic disease history and family history - Menstruation status - Number of deliveries - Fasting bloods |
| Shi-Chang 2004, China^103^ | This pilot project in Zhejiang Province, China, aimed at improving the nutrition and health status of students, school personnel and parents, and developing a model project for nutrition interventions for the development of health- promoting schools in China. | Intervention, Pre-post. *Questionnaire* | Inservice*,  Primary, Secondary | - Dietary and hygienic habits - Nutrition knowledge and attitudes |  | - Knowledge and attitudes to other health problems |
| Sirajuddin 2021, Indonesia^104^ | To determine the effect of rice bran milk on fasting blood glucose levels and body weight of primary school teachers in Makassar City. | Intervention, Quasi experimental. *Clinical, 24-Hour Recall, Anthropometric* | Inservice,  Primary, Elementary | - Dietary intake - Waist circumference - Weight |  | - Fasting bloods |
| Story 2000, United States of America^105^ | Process evaluation was conducted by using surveys and classroom and lunchroom observations to assess the characteristics of teachers and food service staff, the degree the intervention was implemented as intended, and external factors that may have affected the program results. | Intervention, Pre-post. *Questionnaire, Observations* | Inservice*,  Elementary | - Importance of eating fruit and vegetables - Dietary intake | - Confidence in teaching the curriculum |  |

Legend: *Included non-teacher participants, ^Direct excerpt from source paper, V Description of validity testing provided, R Description of reliability testing provided.

**Reference list**

1. Al-Gelban KS. Dietary habits and exercise practices among the students of a Saudi teachers' training college. Saudi Med J. 2008;29(5):754-9.

2. Ates H. Elementary school teachers’ behavioral intentions for healthy nutrition. Health Educ. 2019;119(2):133-49.

3. Both J, Borgatto A, Sonoo C, Lemos C, Ciampolini V, Vieira J. Multiple jobholding associated with the wellbeing of physical education teachers in Southern Brazil. Educación Fisica y Deporte. 2016;35:1-14.

4. Both J, Borgatto A, Lemos C, Ciampolini V, Vieira J. Physical education teachers’ wellbeing and its relation with gender. Motricidade. 2017;13:23-32.

5. Chen YH, Yeh CY, Lai YM, Shyu ML, Huang KC, Chiou HY. Significant effects of implementation of health-promoting schools on schoolteachers' nutrition knowledge and dietary intake in Taiwan. Public Health Nutr. 2010;13(4):579-88.

6. Dalais L, Abrahams Z, Steyn N, Villiers A, Fourie J, Hill J, et al. The association between nutrition and physical activity knowledge and weight status of primary school educators. S Afr J Educ. 2014;34.

7. Delfino LD, Tebar WR, Gil FC, De Souza JM, Romanzini M, Fernandes RA, et al. Association of sedentary behaviour patterns with dietary and lifestyle habits among public school teachers: a cross-sectional study. BMJ Open. 2020;10(1):e034322.

8. Dunn C, Whetstone LM, Kolasa KM, Jayaratne KS, Thomas C, Aggarwal S, et al. Delivering a behavior-change weight management program to teachers and state employees in North Carolina. Am J Health Promot. 2013;27(6):378-83.

9. Frerichs L, Brittin J, Intolubbe-Chmil L, Trowbridge M, Sorensen D, Huang TT. The role of school design in shaping healthy eating-related attitudes, practices, and behaviors among school staff. J Sch Health. 2016;86(1):11-22.

10. Gebretatyos H, Amanuel S, Ghirmai L, Gebreyohannes G, Tesfamariam EH. Effect of health education on healthy nutrition and physical activity among female teachers aged 40-60 years in Asmara, Eritrea: a quasiexperimental study. J Nutr Metab. 2020;2020:5721053.

11. Hartline-Grafton HL, Rose D, Johnson CC, Rice JC, Webber LS. The influence of weekday eating patterns on energy intake and BMI among female elementary school personnel. Obesity (Silver Spring). 2010;18(4):736-42.

12. Hartline-Grafton HL, Rose D, Johnson CC, Rice JC, Webber LS. Are school employees role models of healthful eating? Dietary intake results from the ACTION worksite wellness trial. J Am Diet Assoc. 2009;109(9):1548-56.

13. Hartline-Grafton HL, Rose D, Johnson CC, Rice JC, Webber LS. Energy density of foods, but not beverages, is positively associated with body mass index in adult women. Eur J Clin Nutr. 2009;63(12):1411-8.

14. Hasan N, Hadju V, Jafar N, Thaha R. A relationship between knowledge, attitude, and practice about balanced nutrition guidelines and metabolic syndrome among central obese teachers in Makassar. Indian J Public Health Res Dev. 2019;10:579.

15. Hemati M, Toori M, Shams M, Behroozpour A. Effect of an educational intervention on nutrition literacy in teachers: a short communication. Malays J Nutr. 2020;26:495-500.

16. Husain W, Ashkanani F, Al Dwairji MA. Nutrition knowledge among college of basic education students in Kuwait: a cross-sectional study. J Nutr Metab. 2021;2021:5560714.

17. Rombaldi AJ BT, Canabarro LK, Neutzling MB, Correa LQ Knowledge of physcial education teachers about risk factors for chronic disease in a city on Southern Brazil. Revista Brasileira de Cineantroppmetria e Desempenho Humano. 2012;14(1).

18. Katsagoni CN, Apostolou A, Georgoulis M, Psarra G, Bathrellou E, Filippou C, et al. Schoolteachers’ nutrition knowledge, beliefs, and attitudes before and after an e-learning program. J Nutr Educ Behav. 2019;51(9):1088-98.

19. LeCheminant JD, Merrill RM, Masterson T. Health behaviors and work-related outcomes among school employees. Am J Health Behav. 2015;39(3):345-51.

20. LeCheminant J, Merrill RM, Masterson TD. Changes in behaviors and outcomes among school-based employees in a wellness program. Health Promot Pract. 2017;18(6):895-901.

21. Lemon SC, Liu Q, Magner R, Schneider KL, Pbert L. Development and validation of worksite weight-related social norms surveys. Am J Health Behav. 2013;37(1):122-9.

22. Melville DS, Hammermeister J. Pre-service physical educators: their demographics, wellness practices, and teaching interests. The Physical Educator. 2006;63:69+.

23. Merrill RM, Sloan A. Effectiveness of a health promotion program among employees in a Western United States school district. J Occup Environ Med. 2014;56:639–44.

24. Monica SJ JS, Madhanagopal R. Risk of obesity among female school teachers and its associated health problems. Curr Res Nutr Food Sci. 2018;6(2).

25. Motamedrezaei O, Moodi M, Miri MR, Khodadadi M. The effect of nutrition and food hygieneeducation on the knowledge of female elementary school teachers in city of Ferdows. J Educ Health Promot. 2013;2:10.

26. Mullaney MI, Corish CA, Loxley A. Exploring the nutrition and lifestyle knowledge, attitudes and behaviour of student home economics teachers: baseline findings from a 4-year longitudinal study. Int J Consum Stud. 2008;32(4):314-22.

27. O'Dea JA, Abraham S. Knowledge, beliefs, attitudes, and behaviors related to weight control, eating disorders, and body image in Australian trainee home economics and physical education teachers. J Nutr Educ. 2001;33(6):332-40.

28. Rafiroiu AC EA. Nutrition knowledge, attitudes, and practices among nutrition educators in the South. Am J Health Stud 2005;20(1).

29. Russell-Mayhew S, Ireland A, Peat G. The impact of professional development about weight-related issues for pre-service teachers: a pilot study. Alberta J Educ Res. [Internet]. 2012;58(3):314-29.

30. Schultz NS, Chui KKH, Economos CD, Lichtenstein AH, Volpe SL, Sacheck JM. Impact of physical activity, diet quality and stress on cardiometabolic health in school employees. Prev Med Rep. 2020;20:101243.

31. Thaha RM, Hasan N, Hadju V, Jafar N, Muhiddin S, Maria IL. Measuring self-regulation after nutrition education modules with Self Determination Theory (SDT) intervention among teachers with or at risk metabolic syndrome. Gac Sanit. 2021;35 Suppl 1:S83-s6.

32. Vio F, Yañez M, González CG, Fretes G, Salinas J. Teachers' self-perception of their dietary behavior and needs to teach healthy eating habits in the school. J Health Psychol. 2018;23(8):1019-27.

33. Wang D, Stewart D, Chang C. A holistic school-based nutrition program fails to improve teachers’ nutrition-related knowledge, attitudes and behaviour in rural China. Health Educ. 2016;116(5):467-75.

34. Wilkinson C, Prusak K, Johnson T. Self-regulation of physical education teacher education students’ attitudes towards exercise and diet. ICHPER SD Journal of Research. 2013;8:49-54.

35. Woynarowska-Soldan M. Project on school staff health promotion in Poland: the first experiences. Health Education. 2015;115(3/4):405-19.

36. Yager Z, Gray T, Curry C, McLean SA. Body dissatisfaction, excessive exercise, and weight change strategies used by first-year undergraduate students: comparing health and physical education and other education students. J Eat Disord. 2017;5(1):10.

37. Yager Z, O'Dea J. Body image, dieting and disordered eating and activity practices among teacher trainees: implications for school-based health education and obesity prevention programs. Health Educ Res. 2009;24(3):472-82.

38. Arcan C, Hannan PJ, Himes JH, Fulkerson JA, Rock BH, Smyth M, et al. Intervention effects on kindergarten and first-grade teachers' classroom food practices and food-related beliefs in American Indian reservation schools. J Acad Nutr Diet. 2013;113(8):1076-83.

39. Beffa-Negrini PA, Cohen NL, Laus MJ, McLandsborough LA. Development and evaluation of an online, inquiry-based food safety education program for secondary teachers and their students. J Food Sci Educ. 2007;6(4):66-71.

40. Chrisman M, Patel S, Alonzo R. Barriers to and facilitators of using MyPlate nutritional guidelines in K-12 teachers and principals. Health Educ J. 2019;79(2):152-65.

41. Coccia CC, Tamargo J, Macchi AK. Effects of nutrition knowledge, personal health and self-efficacy on food-related teaching practices of elementary school pre-service teachers. Health Educ J. 2020;79(8):974-86.

42. Costello C, Kane M, Davidson PM, Morris WC. Usage of a web-based food safety course to teach high school teachers. J Culin Sci Tech. 2005;4(1):113-22.

43. Eley C, Lundgren PT, Kasza G, Truninger M, Brown C, Hugues VL, et al. Teaching young consumers in Europe: a multicentre qualitative needs assessment with educators on food hygiene and food safety. Perspect Public Health. 2021;142(3):175-83.

44. Elorinne A-L, Eronen L, Pollari M, Hokkanen J, Reijonen H, Murphy J. Investigating home economics teachers' food waste practices and attitudes. J Teach Educ Sustain. 2020;22:6-20.

45. Endres J, Welch T, Perseli T. Use of a computerized kiosk in an assessment of food safety knowledge of high school students and science teachers. J Nutr Educ. 2001;33(1):37-42.

46. Falkenbach D, D'Avila H, Mello E. Knowledge, attitudes and practices of primary school teachers on nutrition and food. International Journal of Nutrology. 2018;11:021-9.

47. Findholt NE, Izumi BT, Shannon J, Nguyen T. Food-related practices and beliefs of rural US elementary and middle school teachers. Rural Remote Health. 2016;16(2):3821.

48. Hamilton L, Goodman L, Roberts L, Dial LA, Pratt M, Musher-Eizenman D. Teacher experience, personal health, and dieting status Is associated with classroom health-related practices and modeling. J Sch Health. 2021;91(2):155-63.

49. Jones AM, Zidenberg-Cherr S. Exploring nutrition education resources and barriers, and nutrition knowledge in teachers in California. J Nutr Educ Behav. 2015;47(2):162-9.

50. Kaschalk-Woods E, Fly AD, Foland EB, Dickinson SL, Chen X. Nutrition curriculum training and implementation improves teachers' self-efficacy, knowledge, and outcome expectations. J Nutr Educ Behav. 2021;53(2):142-50.

51. Kinsler J, Slusser W, Erausquin JT, Thai C, Prelip M. Nutrition knowledge and self-efficacy among classroom teachers from a large urban school district in Los Angeles County. Calif J Health Promot. 2012;10:118-25.

52. Kubik MY LL, Hannan PJ, Story M, Perry CL. Food-related beliefs, eating behavior, and classroom food practices of middle school teachers. J Sch Health. 2002;72(8):339-45.

53. Laguna MC, Hecht AA, Ponce J, Jue T, Brindis CD, Patel AI. Teachers as healthy beverage role models: relationship of student and teacher beverage choices in elementary schools. J Community Health. 2020;45(1):121-7.

54. Metos JM, Sarnoff K, Jordan KC. Teachers' perceived and desired roles in nutrition education. J Sch Health. 2019;89(1):68-76.

55. Molloy CJ, Gandy J, Cunningham C, Slattery G. An exploration of factors that influence the regular consumption of water by Irish primary school children. J Hum Nutr Diet. 2008;21(5):512-5.

56. Wilna O-T, Egal A. Impact of nutrition education on nutrition knowledge of public school educators in South Africa: A pilot study. Health SA Gesondheid. 2012;17.

57. Parker EA, Feinberg TM, Lane HG, Deitch R, Zemanick A, Saksvig BI, et al. Diet quality of elementary and middle school teachers is associated with healthier nutrition-related classroom practices. Prev Med Rep. 2020;18:101087.

58. Perikkou A, Kokkinou E, Panagiotakos DB, Yannakoulia M. Teachers’ readiness to Implement nutrition education programs: beliefs, attitudes, and barriers. J Res Child Educ. 2015;29(2):202-11.

59. Prescott M, Lohse B, Balgopal M, Smith S, Addington R, Cunningham-Sabo L. Teacher well-being attributes are positively associated with teacher perceptions of Fuel for Fun tasting lessons. Top Clin Nutr. 2018;33:272-80.

60. Pivarnik LF, Patnoad MS, Richard NL, Gable RK, Hirsch DW, Madaus J, et al. Assessment of food safety knowledge of high school and transition teachers of special needs students. J Food Sci Educ. 2009;8(1):13-9.

61. Ronto R, Ball L, Pendergast D, Harris ND. Food literacy at secondary schools in Australia. J Sch Health. 2016;86(11):823-31.

62. Rossiter M, Glanville T, Taylor J, Blum I. School food practices of prospective teachers. J Sch Health. 2007;77(10):694-700.

63. Russell-Mayhew S, Nutter S, Ireland A, Gabriele T, Bardick A, Crooks J, et al. Pilot testing a professional development model for preservice teachers in the area of health and weight: feasibility, utility, and efficacy. Adv School Ment Health Promot. 2015;8(3):176-86.

64. Selvam S, Murugesan N, Snehalatha C, Nanditha A, Raghavan A, Simon M, et al. Health education on diabetes and other non-communicable diseases imparted to teachers shows a cascading effect. A study from Southern India. Diabetes Research and Clinical Practice. 2017;125:20-8.

65. Snelling A, Belson SI, Young JL. School health reform: investigating the role of teachers. J Child Nutr Manag. 2012;36.

66. Thompson BM, Ribera KP, Wingenbach GJ, Vestal TA. The relationship between attitudes, knowledge, and demographic variables of high school teachers regarding food irradiation. J Food Sci Educ. 2007;6(2):24-9.

67. Lai Yeung Wai-ling T. Combating deceptive advertisements and labelling on food products – an exploratory study on the perceptions of teachers. Int J Consum Stud. 2004;28(2):117-26.

68. Al-Refaee F, Al-Dhafiri S, Al-Qattan S, Al-Mutairi A, Jaber S, Nassar M. Nutritional knowledge, attitude and practice of high school girls living in Kuwait: a pilot study. Kuwait Med J. 2013;45:118-22.

69. Antwi J, Ohemeng A, Boateng L, Quaidoo E, Bannerman B. Primary school-based nutrition education intervention on nutrition knowledge, attitude and practices among school-age children in Ghana. Glob Health Promot. 2020;27(4):114-22.

70. Hyska J, Burazeri G, Menza V, Dupouy E. Assessing nutritional status and nutrition-related knowledge, attitudes and practices of Albanian schoolchildren to support school food and nutrition policies and programmes. Food Policy. 2020;96:101888.

71. López-Barrón RG, Jiménez-Cruz A, Bacardí-Gascón M. Modifiable environmental obesity risk factors among elementary school children in a Mexico-US border city. Nutr Hosp. 2015;31(5):2047-53.

72. Linnell JD, Smith MH, Briggs M, Brian KM, Scherr RE, Dharmar M, et al. Evaluating the relationships among teacher characteristics, implementation factors, and student outcomes of children participating in an experiential school-based nutrition program. Pedagogy Health Promot. 2016;2(4):256-65.

73. Lozada M, Sánchez-Castillo CP, Cabrera GA, Mata, II, Pichardo-Ontiveros E, Villa AR, et al. School food in Mexican children. Public Health Nutr. 2008;11(9):924-33.

74. Machado S, Ritchie L, Thompson H, Reed A, Castro AI, Neelon M, et al. Multi-pronged intervention to increase secondary student participation in school lunch: design and rationale. Contemp Clin Trials. 2019;78:133-9.

75. McVey G, Tweed S, Blackmore E. Healthy Schools-Healthy Kids: a controlled evaluation of a comprehensive universal eating disorder prevention program. Body Image. 2007;4(2):115-36.

76. Okeyo AP, Seekoe E, de Villiers A, Faber M, Nel JH, Steyn NP. The food and nutrition environment at secondary schools in the Eastern Cape, South Africa as reported by learners. Int J Environ Res Public Health. 2020;17(11).

77. Shah P, Misra A, Gupta N, Hazra D, Gupta R, Seth P, et al. Improvement in nutrition-related knowledge and behaviour of urban Asian Indian school children: Findings from the Medical education for children/Adolescents for Realistic prevention of obesity and diabetes and for healthy aGeing (MARG) intervention study. Br J Nutr. 2010;104:427-36.

78. Talip T, Serudin R, Noor S, Tuah N. Qualitative study of eating habits in Bruneian primary school children. Asia Pac J Clin Nutr. 2017;26(6):1113-8.

79. Berger-Jenkins E, Rausch J, Okah E, Tsao D, Nieto A, Lyda E, et al. Evaluation of a coordinated school-based obesity prevention program in a hispanic community: choosing healthy and active lifestyles for kids/healthy schools healthy families. Am J Health Educ. 2014;45(5):261-70.

80. Gaglianone C, Taddei J, Colugnati F, Magalhães C, Davanço G, de Macedo L, et al. Nutrition education in public elementary schools of São Paulo, Brazil: the reducing risks of illness and death in adulthood project. Rev Nutr. 2006;19.

81. Kupolati MD, MacIntyre UE, Gericke GJ, Becker P. A contextual Nutrition education program improves nutrition knowledge and attitudes of South African teachers and learners. Front Public Health. 2019;7.

82. Laurie SM, Faber M, Maduna MM. Assessment of food gardens as nutrition tool in primary schools in South Africa. South Afr J Clin Nutr. 2017;30(4):80-6.

83. Lee BR, Ko YM, Cho MH, Yoon YR, Kye SH, Park YK. Effects of 12-week vegetarian diet on the nutritional status, stress status and bowel habits in middle school students and teachers. Clin Nutr Res. 2016;5(2):102-11.

84. O’Dea J. Evaluation of nutrition and physical activity knowledge, attitudes, self efficacy and behaviors in teachers and children after implementation of the “Healthy Active Kids” online program in Australian elementary schools. Health. 2016;08:293-303.

85. Barwood D, Smith S, Miller M, Boston J, Masek M, Devine A. Transformational game trial in nutrition education. Aust J Teach Educ. 2020;45:18-29.

86. Canchola AJ, Lacey JV, Jr., Bernstein L, Horn-Ross PL. Dietary patterns and endometrial cancer risk in the California Teachers Study cohort. Cancer Causes Control. 2015;26(4):627-34.

87. Chang ET, Lee VS, Canchola AJ, Clarke CA, Purdie DM, Reynolds P, et al. Diet and risk of ovarian cancer in the California Teachers Study cohort. Am J Epidemiol. 2007;165(7):802-13.

88. Chang ET, Lee VS, Canchola AJ, Dalvi TB, Clarke CA, Reynolds P, et al. Dietary patterns and risk of ovarian cancer in the California Teachers Study cohort. Nutr Cancer. 2008;60(3):285-91.

89. Eng JY, Moy FM, Bulgiba A, Rampal S. Consistency and generalizability of dietary patterns in a multiethnic working Pppulation. J Acad Nutr Diet. 2018;118(7):1249-62.e3.

90. Eng JY, Moy FM, Bulgiba A, Rampal S. Dose-response relationship between western diet and being overweight among teachers in Malaysia. Nutrients. 2020;12(10).

91. Esmaillzadeh A, Kimiagar M, Mehrabi Y, Azadbakht L, Hu FB, Willett WC. Fruit and vegetable intakes, c-reactive protein, and the metabolic syndrome. Am J Clin Nutr. 2006;84(6):1489-97.

92. Esmaillzadeh A, Kimiagar M, Mehrabi Y, Azadbakht L, Hu FB, Willett WC. Dietary patterns, insulin resistance, and prevalence of the metabolic syndrome in women. Am J Clin Nutr. 2007;85(3):910-8.

93. Esmaillzadeh A, Azadbakht L. Legume consumption is inversely associated with serum concentrations of adhesion molecules and inflammatory biomarkers among Iranian women. J Nutr. 2012;142(2):334-9.

94. Haridass V, Ziogas A, Neuhausen SL, Anton-Culver H, Odegaard AO. Diet quality scores inversely associated with postmenopausal breast cancer risk are not associated with premenopausal breast cancer risk in the California Teachers Study. J Nutr. 2018;148(11):1830-7.

95. Horn-Ross PL, Hoggatt KJ, West DW, Krone MR, Stewart SL, Anton H, et al. Recent diet and breast cancer risk: the California Teachers Study (USA). Cancer Causes Control. 2002;13(5):407-15.

96. Jafar N, Hasan N, Hadju V, Thaha RM, Arundhana AI. Improved knowledge, attitudes, and practices of balanced nutrition after educational intervention based on the self-determination theory: an Intervention study in senior school teachers in Makassar City. Open Access Maced J Med Sci. 2020;8(E):228-35.

97. Khatibi N, Shahvazi S, Nadjarzadeh A, Samadi M, Zare F, Salehi-Abargouei A. Empirically derived dietary patterns and serum inflammatory markers in Iranian female teachers: A cross-sectional study. Nutr Diet. 2019;76(4):462-71.

98. Link LB, Canchola AJ, Bernstein L, Clarke CA, Stram DO, Ursin G, et al. Dietary patterns and breast cancer risk in the California Teachers Study cohort. Am J Clin Nutr. 2013;98(6):1524-32.

99. Pacheco LS, Lacey JV, Jr., Martinez ME, Lemus H, Araneta MRG, Sears DD, et al. Sugar-sweetened beverage intake and cardiovascular disease risk in the California Teachers Study. J Am Heart Assoc. 2020;9(10):e014883.

100. Rana L, Alvaro R. Applying a health promoting schools approach to nutrition interventions in schools: key factors for success. Health Promot J Austr. 2010;21(2):106-13.

101. Reynolds P, Hurley SE, Hoggatt K, Anton-Culver H, Bernstein L, Deapen D, et al. Correlates of active and passive smoking in the California Teachers Study cohort. J Womens Health (Larchmt). 2004;13(7):778-90.

102. Sakhaei R, Shahvazi S, Mozaffari-Khosravi H, Samadi M, Khatibi N, Nadjarzadeh A, et al. The dietary approaches to stop hypertension (DASH)-style diet and an alternative mediterranean diet are differently associated with serum inflammatory markers in female adults. Food Nutr Bull. 2018;39(3):361-76.

103. Shi-Chang X, Xin-Wei Z, Shui-Yang X, Shu-Ming T, Sen-Hai Y, Aldinger C, et al. Creating health-promoting schools in China with a focus on nutrition. Health Promotion International. 2004;19(4):409-18.

104. Sirajuddin S M, Salam A. The effect of giving rice bran milk on blood glucose levels and body weight in hyperglycemic primary school teachers in Makassar City. Int J Pharm Res. 2021;13(1).

105. Story M, Mays RW, Bishop DB, Perry CL, Taylor G, Smyth M, et al. 5-a-day power plus: process evaluation of a multicomponent elementary school program to increase fruit and vegetable consumption. Health Educ Behav. 2000;27(2):187-200.
