# Supplementary material for: Addressing schoolteacher food and nutrition-related health and wellbeing: a scoping review of the food and nutrition constructs used across current research
Source: Int J Behav Nutr Phys Act. 2023 Sep 12;20:108. doi: 10.1186/s12966-023-01502-5 (PMC10498614; doi:10.1186/s12966-023-01502-5)
Supplement: Supplementary file 5 — Additional file 5. Personal FN-Summary of Constructs Observed. [file 12966_2023_1502_MOESM5_ESM.docx]

| **Intentions, norms, perceived control, and competence** | **Nutrition practices, resources, and education** | **Food safety practices or knowledge** | **Teacher attitudes and/or eating behaviour at school** | **Dieting status/ weight change behaviours** | **Culinary** | **Disordered eating, practices, attitudes, and behaviours** | **Body image** |
| --- | --- | --- | --- | --- | --- | --- | --- |
| Confidence in ability to eat healthfully^1^ | Nutrition practice (food habits and choices, hygienic measures)^2^ | Food safety practices^3^ | Eating behaviours at school^4^  Teacher eating patterns at school^5^  Prospective teacher eating patterns at school^6^  Teacher school lunches^7^  Teacher eating habits at school^8^ | Dieting status^9^ | Cooking habits and responsibilities^10^ | Disordered eating^11^ | Body satisfaction^11^ |
| Perceptions of healthy eating^12^ | Nutrition and Physical activity patterns^13^ | Food safety behaviours (i.e., how often do they follow key food safety behaviours)^3^ | Beverages consumed at school^14^ | Weight control practices^15^  Currently trying to loose weight^38^  Attempts at weight loss^41^ | Frequency of home meal preparation^16^ | History of Eating disorder^15^  Past and current treatment of eating disorder^29^ | Internalisation of media stereotypes^11^  The figure rating scale^29^ |
| Future food choice intentions score^17^ | Wellness practices  SQ: Are you able to find time in the day for a balanced diet of three or more meals^18^ | Food safety (Questions not provided, practices and confidence in teaching food safety is covered)^19^ | Food composition (school food environment and what foods were available in this space)^20^ | Weight control behaviours^21^ | Average time spent preparing a meal^16^ | Eating attitudes (The EAT survey screening tool)^22,23^ | Body image^15,32^ |
| Intentions  SQ: I will try to eat healthy foods for the next 2 weeks on a regular basis^24^ | Sourcing nutrition information^10^ | Food safety knowledge test ^25^  Food safety knowledge ^26^ | School lunch participation^27^ | Weight change behaviours^22^ | Teachers' culinary habits^28^ | Disordered eating^29^ | Body size perceptions^21^  Body weight perceptions^29^ |
| Subjective norms^24^ | No' of college nutrition courses taken^16^ | Food safety^30^ | Classroom practices^31^ | Weight change behaviours, includes disordered eating^32^ | Cooking attitudes^16^ | Knowledge, Behaviour, Attitudes about weight control and eating disorders^15^ | Body satisfaction^22,23^ |
| Personal norms  SQ: I feel guilty if I do not eat healthy foods^24^ | Improvement in lifestyle practices (including healthy eating)^33^ |  | Teacher nutrition attitudes and beliefs^34^ | Dieting behaviours^29^ | Self-efficacy and attitudes towards food literacy^35^ | Anti-fat attitudes^23^ |  |
| Self-identity  SQ: I think of myself as someone healthy^24^ | Nutritional characteristics^36^ |  | Attitudes and perceptions of school lunch^27^ | Weight perceptions^40^  Perceived weight^41^ | Dietary habits^37^ |  |  |
| Worksite related social norms and social support for Eating^38^ |  |  | Teacher Characteristics^39^ |  |  |  |  |

***Note: All construct names and sample questions are direct excerpts from the referenced included review papers.***

**Reference list**

1. Dunn C, Whetstone LM, Kolasa KM, Jayaratne KS, Thomas C, Aggarwal S, et al. Delivering a behavior-change weight management program to teachers and state employees in North Carolina. Am J Health Promot. 2013;27(6):378-83.

2. Al-Refaee F, Al-Dhafiri S, Al-Qattan S, Al-Mutairi A, Jaber S, Nassar M. Nutritional knowledge, attitude and practice of high school girls living in Kuwait: a pilot study. Kuwait Med J. 2013;45:118-22.

3. Beffa-Negrini PA, Cohen NL, Laus MJ, McLandsborough LA. Development and evaluation of an online, inquiry-based food safety education program for secondary teachers and their students. J Food Sci Educ. 2007;6(4):66-71.

4. Findholt NE, Izumi BT, Shannon J, Nguyen T. Food-related practices and beliefs of rural US elementary and middle school teachers. Rural Remote Health. 2016;16(2):3821.

5. Kubik MY LL, Hannan PJ, Story M, Perry CL. Food-related beliefs, eating behavior, and classroom food practices of middle school teachers. J Sch Health. 2002;72(8):339-45.

6. Rossiter M, Glanville T, Taylor J, Blum I. School food practices of prospective teachers. J Sch Health. 2007;77(10):694-700.

7. Lozada M, Sánchez-Castillo CP, Cabrera GA, Mata, II, Pichardo-Ontiveros E, Villa AR, et al. School food in Mexican children. Public Health Nutr. 2008;11(9):924-33.

8. Arcan C, Hannan PJ, Himes JH, Fulkerson JA, Rock BH, Smyth M, et al. Intervention effects on kindergarten and first-grade teachers' classroom food practices and food-related beliefs in American Indian reservation schools. J Acad Nutr Diet. 2013;113(8):1076-83.

9. Hamilton L, Goodman L, Roberts L, Dial LA, Pratt M, Musher-Eizenman D. Teacher experience, personal health, and dieting status is associated with classroom health-related practices and modeling. J Sch Health. 2021;91(2):155-63.

10. Husain W, Ashkanani F, Al Dwairji MA. Nutrition knowledge among college of basic education students in Kuwait: a cross-sectional study. J Nutr Metabol. 2021;2021:5560714.

11. McVey G, Tweed S, Blackmore E. Healthy Schools-Healthy Kids: a controlled evaluation of a comprehensive universal eating disorder prevention program. Body Image. 2007;4(2):115-36.

12. Talip T, Serudin R, Noor S, Tuah N. Qualitative study of eating habits in Bruneian primary school children. Asia Pac J Clin Nutr. 2017;26(6):1113-8.

13. Snelling A, Belson SI, Young JL. School health reform: investigating the role of teachers. J Child Nutr Manag. 2012;36.

14. Laguna MC, Hecht AA, Ponce J, Jue T, Brindis CD, Patel AI. Teachers as healthy beverage role models: relationship of student and teacher beverage choices in elementary schools. J Community Health. 2020;45(1):121-7.

15. O'Dea JA, Abraham S. Knowledge, beliefs, attitudes, and behaviors related to weight control, eating disorders, and body image in Australian trainee home economics and physical education teachers. J Nutr Educ. 2001;33(6):332-40.

16. Prescott M, Lohse B, Balgopal M, Smith S, Addington R, Cunningham-Sabo L. Teacher well-being attributes are positively associated with teacher perceptions of Fuel for Fun tasting lessons. Topics in Clinical Nutrition. 2018;33:272-80.

17. Barwood D, Smith S, Miller M, Boston J, Masek M, Devine A. Transformational game trial in nutrition education. Aust J Teach Educ. 2020;45:18-29.

18. Melville DS, Hammermeister J. Pre-service physical educators: their demographics, wellness practices, and teaching interests. The Physical Educator. 2006;63:69+.

19. Eley C, Lundgren PT, Kasza G, Truninger M, Brown C, Hugues VL, et al. Teaching young consumers in Europe: a multicentre qualitative needs assessment with educators on food hygiene and food safety. Perspect Public Health. 2021;142(3):175-83.

20. López-Barrón RG, Jiménez-Cruz A, Bacardí-Gascón M. Modifiable environmental obesity risk factors among elementary school children in a Mexico-US border city. Nutr Hosp. 2015;31(5):2047-53.

21. Rafiroiu AC EA. Nutrition knowledge, attitudes, and practices among nutrition educators in the south. Am J Health Stud 2005;20(1).

22. Russell-Mayhew S, Ireland A, Peat G. The impact of professional development about weight-related issues for pre-service teachers: a pilot study. Alberta J Educ Res. [Internet]. 2012;58(3):314-29.

23. Russell-Mayhew S, Nutter S, Ireland A, Gabriele T, Bardick A, Crooks J, et al. Pilot testing a professional development model for preservice teachers in the area of health and weight: feasibility, utility, and efficacy. Advances in School Mental Health Promotion. 2015;8(3):176-86.

24. Ates H. Elementary school teachers’ behavioral intentions for healthy nutrition. Health Educ. 2019;119(2):133-49.

25. Costello C, Kane M, Davidson PM, Morris WC. Usage of a web-based food safety course to teach high school teachers. J Culin Sci Tech. 2005;4(1):113-22.

26. Pivarnik LF, Patnoad MS, Richard NL, Gable RK, Hirsch DW, Madaus J, et al. Assessment of food safety knowledge of high school and transition teachers of special needs students. J Food Sci Educ. 2009;8(1):13-9.

27. Machado S, Ritchie L, Thompson H, Reed A, Castro AI, Neelon M, et al. Multi-pronged intervention to increase secondary student participation in school lunch: Design and rationale. Contemp Clin Trials. 2019;78:133-9.

28. Vio F, Yañez M, González CG, Fretes G, Salinas J. Teachers' self-perception of their dietary behavior and needs to teach healthy eating habits in the school. J Health Psychol. 2018;23(8):1019-27.

29. Yager Z, O'Dea J. Body image, dieting and disordered eating and activity practices among teacher trainees: implications for school-based health education and obesity prevention programs. Health Educ Res. 2009;24(3):472-82.

30. Endres J, Welch T, Perseli T. Use of a computerized kiosk in an assessment of food safety knowledge of high school students and science teachers. J Nutr Educ. 2001;33(1):37-42.

31. Parker EA, Feinberg TM, Lane HG, Deitch R, Zemanick A, Saksvig BI, et al. Diet quality of elementary and middle school teachers is associated with healthier nutrition-related classroom practices. Prev Med Rep. 2020;18:101087.

32. Yager Z, Gray T, Curry C, McLean SA. Body dissatisfaction, excessive exercise, and weight change strategies used by first-year undergraduate students: comparing health and physical education and other education students. Journal of Eating Disorders. 2017;5(1):10.

33. Selvam S, Murugesan N, Snehalatha C, Nanditha A, Raghavan A, Simon M, et al. Health education on diabetes and other non-communicable diseases imparted to teachers shows a cascading effect. A study from Southern India. Diabetes Research and Clinical Practice. 2017;125:20-8.

34. Katsagoni CN, Apostolou A, Georgoulis M, Psarra G, Bathrellou E, Filippou C, et al. Schoolteachers’ nutrition knowledge, beliefs, and attitudes before and after an e-learning program. J Nutr Educ Behav. 2019;51(9):1088-98.

35. Ronto R, Ball L, Pendergast D, Harris ND. Food literacy at secondary schools in Australia. J Sch Health. 2016;86(11):823-31.

36. Rombaldi AJ BT, Canabarro LK, Neutzling MB, Correa LQ. Knowledge of physcial education teachers about risk factors for chronic disease in a city on southern Brazil. Revista Brasileira de Cineantroppmetria e Desempenho Humano. 2012;14(1).

37. Al-Gelban KS. Dietary habits and exercise practices among the students of a Saudi teachers' training college. Saudi Med J. 2008;29(5):754-9.

38. Lemon SC, Liu Q, Magner R, Schneider KL, Pbert L. Development and validation of worksite weight-related social norms surveys. Am J Health Behav. 2013;37(1):122-9.

39. Story M, Mays RW, Bishop DB, Perry CL, Taylor G, Smyth M, et al. 5-a-day power plus: process evaluation of a multicomponent elementary school program to increase fruit and vegetable consumption. Health Educ Behav. 2000;27(2):187-200.

40. Chen YH, Yeh CY, Lai YM, Shyu ML, Huang KC, Chiou HY. Significant effects of implementation of health-promoting schools on schoolteachers' nutrition knowledge and dietary intake in Taiwan. Public Health Nutr. 2010;13(4):579-88.

41. Dalais L, Abrahams Z, Steyn N, Villiers A, Fourie J, Hill J, et al. The association between nutrition and physical activity knowledge and weight status of primary school educators. S Afr J Educ. 2014;34.
