# Supplementary material for: Addressing schoolteacher food and nutrition-related health and wellbeing: a scoping review of the food and nutrition constructs used across current research
Source: Int J Behav Nutr Phys Act. 2023 Sep 12;20:108. doi: 10.1186/s12966-023-01502-5 (PMC10498614; doi:10.1186/s12966-023-01502-5)
Supplement: Supplementary file 6 — Additional file 6. Professional FN-Summary of Constructs Observed. [file 12966_2023_1502_MOESM6_ESM.docx]

| **Classroom practices and role modelling** | **School practices, attitudes, beliefs** | **Education self-efficacy** | **Education intentions** | **Professional development /or resources used** | **Student focused nutrition knowledge** | **Barriers to teaching nutrition** | **Teaching characteristics and/or fidelity focus** |
| --- | --- | --- | --- | --- | --- | --- | --- |
| Classroom food related practices^1^  Classroom food practices^2,3,4^ | School-wide food practices^1^ | Food safety teaching self-efficacy and confidence/or comfort in teaching Food safety^5^ | Intention to teach food safety in coming year^5^ | Food and nutrition focused professional development completed in the past 3 years^6^ | Nutrition knowledge focused on children and teaching^2^ | Barriers to nutrition education teaching^7^ | Teaching characteristics^8^ |
| Food waste pedagogical practices^9^ | Attitudes to school food environment and food and nutrition education^10^ | Nutrition teaching self-efficacy^2,11,12,13,14^ | Integration of food gardening into the curriculum^15^ | Use of nutrition resources particularly MyPlate^16^ | Nutrition knowledge: Current dietary recommendations for children^17^ | Barriers to nutrition education delivery^18^ | Teachers self-reported degree of completion of the program^13^ |
| Teacher school Food and nutrition practices^10^ | School food environment^4,19,20,21^  Beliefs regarding the school-food environment^1,3^  Beliefs and attitudes about the school food environment^2^ | Food safety teaching self-efficacy^22^ | Teacher readiness to take and accomplish^23^ | Resources used to teach food safety^22^ | Knowledge attitudes and behaviours about suitable weight control advice for students^24^ | Attitudes and barriers to implementing health programs^23^ | Teacher experiences with Fuel for Fun lessons^25^ |
| Classroom food practices and modelling^26^ | The nutritional environment and related context in their respective schools^27^ | Confidence in teaching about diet and nutrition in the classroom^28^ |  | Resources used by teachers to plan nutrition lessons^7^ | Nutrition knowledge regarding student requirements^3^ |  | Rating of the lesson delivery of Fuel for Fun educators^25^ |
| Classroom nutrition practices^14^  Intended classroom food practices^21^  Nutrition-related classroom practices^29^ | Perceptions on ways to improve water consumption in students^30^ | Self-efficacy to participate in weight and body image related contexts within the school environment^31^ |  | Nutrition education practices and resources used or needed, nutrition education topics to be included in the syllabus^32^ | Nutrition knowledge including child nutrition and child health^27^ |  | Curriculum audit^33^ |
| Opinions and practices of water in classroom^34^ | Food policy at school^35^  Food related school policy^1^ | Teachers’ beliefs and self-efficacy on school health education, perceived role in teaching health^36^ |  |  | Nutrition knowledge student nutrition and dietary intake requirements^12^ |  |  |
| Food preparation practices in the classroom^33^ | Perception of the school climate^37^ | Confidence in teaching the curriculum^38^ |  |  |  |  |  |
| Role modelling capability and  classroom food practices^19^  Modelling and encouraging of healthy eating behaviours^39^ | Perceived role of school cafeteria^40^ |  |  |  |  |  |  |
| Beverage consumption in front of students and in the classroom^30^ | Importance of aspects of food literacy^20^ |  |  |  |  |  |  |
|  | Perceptions of what healthy eating should be for students^41^ |  |  |  |  |  |  |
|  | Attitudes and beliefs about teacher role in nutrition education^14^ |  |  |  |  |  |  |
|  | Beliefs and attitudes regarding nutrition^18^ |  |  |  |  |  |  |
|  | Food safety attitudes^33^ |  |  |  |  |  |  |
|  | Teaching attitudes and modelling^42^ |  |  |  |  |  |  |

***Note: All construct names and sample questions are direct excerpts from the referenced included review papers.***

**Reference List**

1. Arcan C, Hannan PJ, Himes JH, Fulkerson JA, Rock BH, Smyth M, et al. Intervention effects on kindergarten and first-grade teachers' classroom food practices and food-related beliefs in American Indian reservation schools. J Acad Nutr Diet. 2013;113(8):1076-83.

2. Coccia CC, Tamargo J, Macchi AK. Effects of nutrition knowledge, personal health and self-efficacy on food-related teaching practices of elementary school pre-service teachers. Health Educ J. 2020;79(8):974-86.

3. Findholt NE, Izumi BT, Shannon J, Nguyen T. Food-related practices and beliefs of rural US elementary and middle school teachers. Rural Remote Health. 2016;16(2):3821.

4. Kubik MY LL, Hannan PJ, Story M, Perry CL. Food-related beliefs, eating behavior, and classroom food practices of middle school teachers. J Sch Health. 2002;72(8):339-45.

5. Beffa-Negrini PA, Cohen NL, Laus MJ, McLandsborough LA. Development and evaluation of an online, inquiry-based food safety education program for secondary teachers and their students. J Food Sci Educ. 2007;6(4):66-71.

6. Chen YH, Yeh CY, Lai YM, Shyu ML, Huang KC, Chiou HY. Significant effects of implementation of health-promoting schools on schoolteachers' nutrition knowledge and dietary intake in Taiwan. Public Health Nutr. 2010;13(4):579-88.

7. Jones AM, Zidenberg-Cherr S. Exploring nutrition education resources and barriers, and nutrition knowledge in teachers in California. J Nutr Educ Behav. 2015;47(2):162-9.

8. Rombaldi AJ BT, Canabarro LK, Neutzling MB, Correa LQ Knowledge of physcial education teachers about risk factors for chronic disease in a city on Southern Brazil. Revista Brasileira de Cineantroppmetria e Desempenho Humano. 2012;14(1).

9. Elorinne A-L, Eronen L, Pollari M, Hokkanen J, Reijonen H, Murphy J. Investigating home economics teachers' food waste practices and attitudes. J Teach Educ Sustain. 2020;22:6-20.

10. Falkenbach D, D'Avila H, Mello E. Knowledge, attitudes and practices of primary school teachers on nutrition and food. International Journal of Nutrology. 2018;11:021-9.

11. Kaschalk-Woods E, Fly AD, Foland EB, Dickinson SL, Chen X. Nutrition curriculum training and implementation improves teachers' self-efficacy, knowledge, and outcome expectations. J Nutr Educ Behav. 2021;53(2):142-50.

12. Kinsler J, Slusser W, Erausquin JT, Thai C, Prelip M. Nutrition knowledge and self-efficacy among classroom teachers from a large urban school district in Los Angeles County. Calif J Health Promot. 2012;10:118-25.

13. Linnell JD, Smith MH, Briggs M, Brian KM, Scherr RE, Dharmar M, et al. Evaluating the relationships among teacher characteristics, implementation factors, and student outcomes of children participating in an experiential school-based nutrition program. Pedagogy Health Promot. 2016;2(4):256-65.

14. Metos JM, Sarnoff K, Jordan KC. Teachers' perceived and desired roles in nutrition education. J Sch Health. 2019;89(1):68-76.

15. Laurie SM, Faber M, Maduna MM. Assessment of food gardens as nutrition tool in primary schools in South Africa. South Afr J Clin Nutr. 2017;30(4):80-6.

16. Chrisman M, Patel S, Alonzo R. Barriers to and facilitators of using MyPlate nutritional guidelines in K-12 teachers and principals. Health Educ J. 2019;79(2):152-65.

17. Kupolati MD, MacIntyre UE, Gericke GJ, Becker P. A contextual nutrition education program improves nutrition knowledge and attitudes of South African teachers and learners. Front Public Health. 2019;7.

18. Katsagoni CN, Apostolou A, Georgoulis M, Psarra G, Bathrellou E, Filippou C, et al. Schoolteachers’ nutrition knowledge, beliefs, and attitudes before and after an e-learning program. J Nutr Educ Behav. 2019;51(9):1088-98.

19. Frerichs L, Brittin J, Intolubbe-Chmil L, Trowbridge M, Sorensen D, Huang TT. The role of school design in shaping healthy eating-related attitudes, practices, and behaviors among school staff. J Sch Health. 2016;86(1):11-22.

20. Ronto R, Ball L, Pendergast D, Harris ND. Food literacy at secondary schools in Australia. J Sch Health. 2016;86(11):823-31.

21. Rossiter M, Glanville T, Taylor J, Blum I. School food practices of prospective teachers. J Sch Health. 2007;77(10):694-700.

22. Eley C, Lundgren PT, Kasza G, Truninger M, Brown C, Hugues VL, et al. Teaching young consumers in Europe: a multicentre qualitative needs assessment with educators on food hygiene and food safety. Perspect Public Health. 2021;142(3):175-83.

23. Perikkou A, Kokkinou E, Panagiotakos DB, Yannakoulia M. Teachers’ readiness to Implement nutrition education programs: beliefs, attitudes, and barriers. J Res Child Educ. 2015;29(2):202-11.

24. O'Dea JA, Abraham S. Knowledge, beliefs, attitudes, and behaviors related to weight control, eating disorders, and body image in Australian trainee home economics and physical education teachers. J Nutr Educ. 2001;33(6):332-40.

25. Prescott M, Lohse B, Balgopal M, Smith S, Addington R, Cunningham-Sabo L. Teacher well-being attributes are positively associated with teacher perceptions of Fuel for Fun tasting lessons. Top Clin Nutr. 2018;33:272-80.

26. Hamilton L, Goodman L, Roberts L, Dial LA, Pratt M, Musher-Eizenman D. Teacher experience, personal health, and dieting status Is associated with classroom health-related practices and modeling. J Sch Health. 2021;91(2):155-63.

27. Hyska J, Burazeri G, Menza V, Dupouy E. Assessing nutritional status and nutrition-related knowledge, attitudes and practices of Albanian schoolchildren to support school food and nutrition policies and programmes. Food Policy. 2020;96:101888.

28. O’Dea J. Evaluation of nutrition and physical activity knowledge, attitudes, self efficacy and behaviors in teachers and children after implementation of the “Healthy Active Kids” online program in Australian elementary schools. Health. 2016;08:293-303.

29. Parker EA, Feinberg TM, Lane HG, Deitch R, Zemanick A, Saksvig BI, et al. Diet quality of elementary and middle school teachers is associated with healthier nutrition-related classroom practices. Prev Med Rep. 2020;18:101087.

30. Laguna MC, Hecht AA, Ponce J, Jue T, Brindis CD, Patel AI. Teachers as healthy beverage role models: relationship of student and teacher beverage choices in elementary schools. J Community Health. 2020;45(1):121-7.

31. Russell-Mayhew S, Nutter S, Ireland A, Gabriele T, Bardick A, Crooks J, et al. Pilot testing a professional development model for preservice teachers in the area of health and weight: feasibility, utility, and efficacy. Adv School Ment Health Promot. 2015;8(3):176-86.

32. Wilna O-T, Egal A. Impact of nutrition education on nutrition knowledge of public school educators in South Africa: A pilot study. Health SA Gesondheid. 2012;17.

33. Pivarnik LF, Patnoad MS, Richard NL, Gable RK, Hirsch DW, Madaus J, et al. Assessment of food safety knowledge of high school and transition teachers of special needs students. J Food Sci Educ. 2009;8(1):13-9.

34. Molloy CJ, Gandy J, Cunningham C, Slattery G. An exploration of factors that influence the regular consumption of water by Irish primary school children. J Hum Nutr Diet. 2008;21(5):512-5.

35. Lozada M, Sánchez-Castillo CP, Cabrera GA, Mata, II, Pichardo-Ontiveros E, Villa AR, et al. School food in Mexican children. Public Health Nutr. 2008;11(9):924-33.

36. Snelling A, Belson SI, Young JL. School health reform: investigating the role of teachers. J Child Nutr Manag. 2012;36.

37. McVey G, Tweed S, Blackmore E. Healthy Schools-Healthy Kids: a controlled evaluation of a comprehensive universal eating disorder prevention program. Body Image. 2007;4(2):115-36.

38. Story M, Mays RW, Bishop DB, Perry CL, Taylor G, Smyth M, et al. 5-a-day power plus: process evaluation of a multicomponent elementary school program to increase fruit and vegetable consumption. Health Educ Behav. 2000;27(2):187-200.

39. Machado S, Ritchie L, Thompson H, Reed A, Castro AI, Neelon M, et al. Multi-pronged intervention to increase secondary student participation in school lunch: design and rationale. Contemp Clin Trials. 2019;78:133-9.

40. Rafiroiu AC EA. Nutrition knowledge, attitudes, and practices among nutrition educators in the South. Am J Health Stud 2005;20(1).

41. Talip T, Serudin R, Noor S, Tuah N. Qualitative study of eating habits in Bruneian primary school children. Asia Pac J Clin Nutr. 2017;26(6):1113-8.

42. Gaglianone C, Taddei J, Colugnati F, Magalhães C, Davanço G, de Macedo L, et al. Nutrition education in public elementary schools of São Paulo, Brazil: the reducing risks of illness and death in adulthood project. Rev Nutr. 2006;19.
