# Supplementary material for: Addressing schoolteacher food and nutrition-related health and wellbeing: a scoping review of the food and nutrition constructs used across current research
Source: Int J Behav Nutr Phys Act. 2023 Sep 12;20:108. doi: 10.1186/s12966-023-01502-5 (PMC10498614; doi:10.1186/s12966-023-01502-5)
Supplement: Supplementary file 7 — Additional file 7. Main Constructs. [file 12966_2023_1502_MOESM7_ESM.docx]

| Personal Food and Nutrition Factor: **Dietary Assessment**  *Overview of constructs, and descriptions* | | |
| --- | --- | --- |
| **Title or name of construct**  *(As identified in the reference paper)* | **Description of construct content/or methodology**  *(As provided in the reference paper)* | **Reference (s)** |
| Dietary intake | Measured as frequency and type of food consumed weekly | 1 |
|  | One week food diary | 2 |
|  | Intake of salty foods, type of diet (vegetarian, non- vegetarian, ovo-vegetarian), junk food consumption | 3 |
|  | Short food frequency questions of selected food groups | 4 |
|  | Food frequency questionnaire, including:   - semi-qualitative food frequency questionnaire - short item food frequency questionnaire | 5, 6, 7, 8, 9, 10, 11, 12, 13, 14, 15, 16, 17, 18, 19, 20, 21, 22 |
|  | Fruit and vegetable   - Screener - Frequency of consumption/intake - Frequency with meals or as snacks | 6, 23, 24, 25, 26, 27, 28 |
|  | Fat screener | 29, 30 |
|  | 24-hour recall (Dietitian Conducted) | 31, 32, 33 |
|  | 24-hour recall (Analysed with a Nutri-survey) | 34 |
|  | Automated 24-hour recall | 35, 36 |
| Dietary pattern analysis | *Including: additional food or beverage group consumption exploration*   - Legume consumption and energy intake - Assessment of sugar sweetened beverage intake - Energy density of foods and beverages - Eating occasion frequency (meal and snack frequency) - Energy intake | 7, 8, 9, 10, 13, 14, 15, 17, 20, 21, 31 |
| Dietary index or quality score calculated | Including   - Dietary Approaches to Stop Hypertension diet score/index - Mediterranean diet score/index - Healthy eating index 2015, American healthy eating index 2010 - Paleo index-adherence to a hunter gathers diet of the paleolithic era | 9, 18, 36 |
| Dietary habits | Food frequency questionnaire about weekly frequency of selected food groups | 37 |
| Behaviours | Adapted from a published food frequency questionnaire focused on how often individuals ate foods in eight major food group (grains, proteins, dairy, fruits, vegetables, fast food, sweets, and other snacks) | 38 |
| Vegetarian diet | *Not assessed but supplied as part of the intervention.*   - 12-week vegetarian diet provided for all school meals, with education and support provided on increasing vegetarian foods in at home meals. | 39 |
| Nutrition practice | Measured by asking questions on food habits and choices, including hygiene | 40 |
| Nutrition patterns | Assessed the consumption of soda, fruit, vegetables, and water. | 41 |

***Note: All construct names and sample questions are direct excerpts from the referenced included review papers.***

| Personal Food and Nutrition Factor: **Nutrition Knowledge**  *Overview of constructs, and descriptions* | | | |
| --- | --- | --- | --- |
| **Title or name of construct**  *(As identified in the reference paper)* | **Description of construct content**  *(As provided in the reference paper, SQ: Sample Questions)* | **Includes student focused questions** (Y/N/Unknown) | **Reference (s)** |
| **Nutrition Knowledge** | Content included:   - Food groups - Fruit and vegetable servings   **SQ:** Which of the following are food groups according to MyPyramid (grains/ vegetables/ fruits/ milk/ meat and beans/ all the above)? | Y | 42 |
|  | Addressed concepts from the 2015-2020 dietary guidelines for Americans  Multiple choice, True/False, or fill the blank formats.  **SQ:** How much of your plate should be a combination of fruit and vegetables? | N | 43 |
|  | Created with reference to the South African food based dietary guidelines | N | 44 |
|  | SQ: Fruit drinks count as a fruit serving | Y | 45 |
|  | Sections included:   - Familiarity with my pyramid - Nutrient content of foods - Diet disease relationship | N | 46 |
|  | Content included:   - Food and nutrient function - Food choices - Nutrient deficiency - Sources of nutrients, food, and energy | N | 47 |
|  | Importance of breakfast intake   - Role of food in the body - Main resources of energy supply in body - Intake rate of energy supply in body - Intake rate of milk and dairy for calcium - Plus, five questions on food hygiene | Y | 48 |
|  | True/False style questions in 6 categories:   - Food classification - Nutrient functions - Weight control - Food safety - Recommended daily consumption in childhood - Results from a 2001 national study on schoolchildren’s dietary intake. | Y | 5 |
|  | Content included:   - Good hydration - Obesity assessment - Role of breakfast - Fast food consumption - Nutritional requirements - Nutritional status - Food safety | Y | 49 |
|  | Four areas:   - Awareness of dietary recommendation - Sources of nutrients - Everyday healthy food choices - Diet-disease relationship | N | 50 |
|  | **SQ:** Eating more fruits and vegetables will help you increase fibre in your diet. | N | 51 |
|  | Content included:   - Five food groups - Key nutrients - The Australian guide to Healthy Eating - Food labelling laws | N | 52 |
|  | Content included:   - Nutrition needs - Dietary guidelines for Americans - Healthy meal and snack choices - Nutrition and health fitness - Food safety, sanitation - Self-responsible choices for food intake   **SQ:** Which of the following groups would improve a breakfast of orange juice, whole wheat toast and low-fat milk? | N | 53 |
|  | Content included:   - The main function of necessary nutrients - Which food was richer in certain nutrients - Symptoms and causes of food poisoning | N | 12 |
|  | No content description provided | Unknown | 38,54 |
| **Nutrition and related lifestyle knowledge** | Content included:   - Food groups and constitutes - Current dietary advice - Dietary knowledge about obesity | N | 2 |
| **Nutrition knowledge and attitudes** | Assessed knowledge of and attitudes towards nutrition.  No content description provided for teacher survey. | Unknown | 55 |
| **Self-rated perception of nutrition knowledge** | *Nutrition knowledge not assessed beyond self-rating score of participants before and after; education provided on:*  Basic nutrition included:   - Role of nutrition in health - Foods and nutrition composition   Healthy eating habits for children, including:   - What a good diet constitutes - Dietary needs of children - The South African food-based dietary guidelines - Meal planning - Food portion sizes | Y | 56 |
| **Knowledge about nutrition** | Three areas covered:   - Dietary recommendations - Nutrients - Health benefits | N | 57 |
| **Knowledge, attitudes, and practices about balanced nutrition** | Questions all provided together in a table within the paper, unclear which questions related to knowledge construct, each construct Knowledge, attitudes, and practices were provided with a separate score of low, moderate, or high.  SQ: Eat plenty of vegetables and fruit | N | 58 |
| **Knowledge about nutrition and food** | SQ: Foods with high concentration of carbohydrates (such as rice, pasta, bread, polenta, potato, cassava) are the basis of healthy eating and the main component of most meals) | N | 59 |
| **Food and nutrition knowledge and perceptions** | Self-perceived knowledge of healthy meals and healthy food choices | Y | 60 |
| **Knowledge of nutrients score** | No content description provided | N | 40 |
| **Knowledge of nutrients function score** | No content description provided | N | 40 |
| **Nutrition knowledge index** | Content included:   - Soft drink intake - Importance of milk products - The quality of fruit servings | Y | 30 |
| **Nutrition knowledge and Behaviour** | SQ: Are carbohydrates the main energy source in out diet | N | 61 |
| **Food advertisement knowledge and behaviour** | SQ: I believe in the claims made by functional food advertisements | N | 62 |
| **Knowledge on drinks, water and hydration, fluid recommendations for adults (and children)** | Content included:   - Opinions on drink suitability - Drinks and health - Nutrition recommendations on fluids - Dehydration signs and symptoms | Y | 63 |
| **Knowledge** | Five categories   - Current dietary recommendations for children - Sources of nutrients - Diet-disease relationship - Food processing - Food hygiene | Y | 64 |
| **Training in nutrition and knowledge of the food based dietary guidelines** | Questions were based on recommendations of the food based dietary guidelines in South Africa | Unknown | 65 |
| **Nutrition literacy** | The evaluation instrument of nutritional literacy on adults (EINLA) measure was used.  Content included:   - Number of servings - Numerical literacy - Reading food label | N | 66 |
| **Digestion knowledge score** | SQ: In which part of the digestive tract does most carbohydrate digestion occur? | N | 67 |
| **Healthy food choices knowledge score** | SQ: If a person wanted to decrease sugar in their diet what would be the healthiest drink choice? | N | 67 |
| **Body food use knowledge score** | SQ: Which nutrient is essential to provide energy to the brain? | N | 67 |
| **Nutrition knowledge, attitudes, and practices** | Assessed based on 10 balanced nutrition guidelines | N | 68 |

***Note: All construct names and sample questions are direct excerpts from the referenced included review papers.***

| Personal Food and Nutrition Factor: **Food, eating habits and behaviours**  *Overview of constructs, and descriptions* | | |
| --- | --- | --- |
| **Title or name of construct**  *(As identified in the reference paper)* | **Description of construct content**  *(As provided in the reference paper, SQ: Sample Questions)* | **Reference** |
| Eating habits | Frequency of meals consumed during the week i.e., main meals per week: breakfast, lunch, dinner, and snack habits after breakfast, after lunch and after dinner | 1 |
| Eating/dietary habits | Provided as a negative, intermediate, or positive score with description in another reference and in another language. | 69,70 |
| Dietary behaviours   - Subsection of Nutrient intake behaviours | 1. Dietary behaviour in the past 30 days 2. Nutrient intake behaviours including:  - Being concerned about calories while eating - Label reading for nutrient content when purchasing food  1. Extra questions on  - Eating breakfast every day - Drinking water only - Having five servings of vegetables and fruits daily | 5 |
| Practices/Dietary practices | Four broad areas   - Personal dietary habits - Eating habits at school - Classroom food practices - School-wide food practices | 64 |
| Personal health index | SQ   - I limit the amount of high fat foods items I eat - I consider myself in good to excellent health - I am satisfied with my own eating habits - Most days I eat 5 servings of fruit and vegetables | 71, 30, 29, 51, 72, 4 |
| Food habits and eating behaviour | - Attitude to meal timings - Meal skipping and need for snacks - Variation and balance of nutrient in a meal - Proper food, hygiene, and human health | 40 |
| Behaviour | SQ: How often do you eat healthy | 73 |
| Perceived behaviour control | SQ: If I want, I can easily eat healthy foods | 73 |
| Personal health perceptions | Teacher satisfaction in relation to his/her:   - Health - Eating habits and physical activity - Estimate behaviour and consumption of some foods and nutrients (sugars and fats) | 59 |
| Nutrition | Modified version of the "Health promotion lifestyle profile" which included section on practices including:   - Choose a diet low in saturated fat - Limit use of sugar and food containing sugar - Eat (2-4, 3-5) servings of fruit each day - Eat 2-3 servings of milk or milk products each day - Eat 2-3 servings from meat, poultry, fish, dried beans, eggs, and nuts each day | 74 |
| The eating behaviour inventory | Assessed behaviours associated with weight management, assessing both adaptive and mal-adaptive behaviours | 75 |
| Food habits | Content included items on:   - Frequency of meals and snacks - Dieting to lose or gain weight | 76 |
| Teachers’ Eating habits | SQ   - Please, tell us about your personal eating habits? - If you could evaluate your personal eating habits, what grade would you give them from 1 to 7? - Regarding how you eat on workdays, please tell us what meals you eat here at the school (breakfast, lunch, and snack), the amount of time you have and where you eat them? - Tell us briefly how your eating habits on the weekends are? | 77 |
| Self-regulation of diet | Uses the *Treatment Self-regulation Questionnaire (Diet)* to assess eating self-regulation (with the self-determination theory approach) | 78,79 |
| The Positive health behaviour scale | Frequency of practising the five groups of health enhancing behaviours including a nutrition subscale | 80 |
| Dietary habits, eating behaviour | Both terms used in text and results table  SQ   - Number of meals - Number of skipped breakfast per week - Types of food at breakfast - Reason for skipping a meal - Frequency of overeating - Types of snacks - Selective eating - Night eating - Foods of dislike | 39 |
| Dietary and hygienic habits | No description provided | 55 |

***Note: All construct names and sample questions are direct excerpts from the referenced included review papers.***

| Personal Food and Nutrition Factor: **Nutrition Attitudes**  *Overview of constructs, and descriptions* | | |
| --- | --- | --- |
| **Title or name of construct**  *(As identified in the reference paper)* | **Description of construct content**  *(As provided in the reference paper, SQ: Sample Questions)* | **Reference** |
| Nutrition attitudes and practices | Attitude and practices   - Food intake - Diet quality - Food choices - Food and health | 47 |
| Attitudes to food and drinks score | SQ   - I feel more energetic if I eat wholegrain instead of white bread - What I eat and drink can make a difference to my chance of getting a disease like diabetes or cancer - I will have a healthy body if I don’t eat processed snack foods or drinks - I will prevent myself gradually gaining weight if I eat plenty of fruits and vegetables - I will improve my overall health by drinking mainly water | 67 |
| Nutrition attitudes and behaviours | SQ   - Reasons for weight gain: (Runs in the family; Eating too much, eating unhealthy food; Too little exercise; Lack of knowledge) - Barriers to healthy eating: (Busy lifestyle; Cost; Little/no self-discipline; Little/no motivation to change; Little/no knowledge; Little/no cooking skills; No family support; Don’t enjoy healthy food) - Usual eating plan: (3 meals without snacks; 3 meals with snacks; 1-2 meals without snacks; 1-2 meals with snacks; Nibbling with no specific meals) - Healthy lunch taken to school: yes, no | 44 |
| Attitudes | Attitudes towards healthy behaviour  SQ: For me eating healthy is good/bad; useful/harmful; cheap/expensive | 73 |
| Attitudes towards eating fruits and vegetables |  | 81 |
| Attitudes to healthy nutrition and lifestyle | Covers consumer Attitudes on physical activity, body weight and health | 2 |
| Attitudes towards diet and nutrition | SQ: How do you feel about diet and nutrition | 52 |
| Nutrition attitudes | Content includes   - Nutritional needs - Functions of nutrients in maintaining health - Dietary guidelines for Americans - Healthy food choices - Nutrition and scholastic achievement - Self-responsibility for food intake | 53 |
|  | Investigated teachers attitudes towards nutrition, healthy dietary habits, and food with an expired consumption date | 12 |
|  | SQ   - Value attributed to a healthy diet - Barriers to a healthy diet - Self-efficacy for a healthy diet | 38 |
| Food attitudes | SQ   - I believe food borne illness caused from bacteria in meats is a problem in the US - Food that has been irradiated is safe to eat - I would serve irradiated food to my family | 82 |
| Food value orientations | “Wide Range Responsibility” or “Restricted Responsibility” category determined by responses in questionnaire | 83 |
| Food waste attitudes and practices | SQ   - I check refrigerator contents before going shopping - I use leftovers in cooking - I eat foods beyond their best before date | 83 |
| Nutrition attitudes | No description provided | 64 |
| Nutrition attitudes | Attitudes towards   - Meal timings - Meal skipping and need for snacks - Variation and balance of nutrients in a meal - Proper food - Hygiene and human health | 40 |
| Eating competence | Eating attitudes, food acceptance, internal regulation, contextual skills | 84 |

***Note: All construct names and sample questions are direct excerpts from the referenced included review papers.***

**Reference List**

1. Al-Gelban KS. Dietary habits and exercise practices among the students of a Saudi teachers' training college. Saudi Med J. 2008;29(5):754-9.
2. Mullaney MI, Corish CA, Loxley A. Exploring the nutrition and lifestyle knowledge, attitudes and behaviour of student home economics teachers: baseline findings from a 4-year longitudinal study. Int J Consum Stud. 2008;32(4):314-22.
3. Monica SJ JS, Madhanagopal R. Risk of obesity among female school teachers and its associated health problems. Current Research Nutrition Food Science 2018;6(2).
4. Perikkou A, Kokkinou E, Panagiotakos DB, Yannakoulia M. Teachers’ readiness to Implement nutrition education programs: beliefs, attitudes, and barriers. J Res Child Educ. 2015;29(2):202-11.
5. Chen YH, Yeh CY, Lai YM, Shyu ML, Huang KC, Chiou HY. Significant effects of implementation of health-promoting schools on schoolteachers' nutrition knowledge and dietary intake in Taiwan. Public Health Nutr. 2010;13(4):579-88.
6. Esmaillzadeh A, Kimiagar M, Mehrabi Y, Azadbakht L, Hu FB, Willett WC. Fruit and vegetable intakes, c-reactive protein, and the metabolic syndrome. Am J Clin Nutr. 2006;84(6):1489-97.
7. Esmaillzadeh A, Kimiagar M, Mehrabi Y, Azadbakht L, Hu FB, Willett WC. Dietary patterns, insulin resistance, and prevalence of the metabolic syndrome in women. Am J Clin Nutr. 2007;85(3):910-8.
8. Esmaillzadeh A, Azadbakht L. Legume consumption is inversely associated with serum concentrations of adhesion molecules and inflammatory biomarkers among Iranian women. J Nutr. 2012;142(2):334-9.
9. Sakhaei R, Shahvazi S, Mozaffari-Khosravi H, Samadi M, Khatibi N, Nadjarzadeh A, et al. The dietary approaches to stop hypertension (DASH)-style diet and an alternative mediterranean diet are differently associated with serum inflammatory markers in female adults. Food Nutr Bull. 2018;39(3):361-76.
10. Khatibi N, Shahvazi S, Nadjarzadeh A, Samadi M, Zare F, Salehi-Abargouei A. Empirically derived dietary patterns and serum inflammatory markers in Iranian female teachers: a cross-sectional study. Nutr Diet. 2019;76(4):462-71.
11. López-Barrón RG, Jiménez-Cruz A, Bacardí-Gascón M. Modifiable environmental obesity risk factors among elementary school children in a Mexico-US border city. Nutr Hosp. 2015;31(5):2047-53.
12. Wang D, Stewart D, Chang C. A holistic school-based nutrition program fails to improve teachers’ nutrition-related knowledge, attitudes and behaviour in rural China. Health Educ. 2016;116(5):467-75.
13. Eng JY, Moy FM, Bulgiba A, Rampal S. Consistency and generalizability of dietary patterns in a multiethnic working Pppulation. J Acad Nutr Diet. 2018;118(7):1249-62.e3.
14. Eng JY, Moy FM, Bulgiba A, Rampal S. Dose-response relationship between western diet and being overweight among teachers in Malaysia. Nutrients. 2020;12(10).
15. Canchola AJ, Lacey JV, Jr., Bernstein L, Horn-Ross PL. Dietary patterns and endometrial cancer risk in the California Teachers Study cohort. Cancer Causes Control. 2015;26(4):627-34.
16. Chang ET, Lee VS, Canchola AJ, Clarke CA, Purdie DM, Reynolds P, et al. Diet and risk of ovarian cancer in the California Teachers Study cohort. Am J Epidemiol. 2007;165(7):802-13.
17. Chang ET, Lee VS, Canchola AJ, Dalvi TB, Clarke CA, Reynolds P, et al. Dietary patterns and risk of ovarian cancer in the California Teachers Study cohort. Nutr Cancer. 2008;60(3):285-91.
18. Haridass V, Ziogas A, Neuhausen SL, Anton-Culver H, Odegaard AO. Diet quality scores inversely associated with postmenopausal breast cancer risk are not associated with premenopausal breast cancer risk in the California Teachers Study. J Nutr. 2018;148(11):1830-7.
19. Horn-Ross PL, Hoggatt KJ, West DW, Krone MR, Stewart SL, Anton H, et al. Recent diet and breast cancer risk: the California Teachers Study (USA). Cancer Causes Control. 2002;13(5):407-15.
20. Link LB, Canchola AJ, Bernstein L, Clarke CA, Stram DO, Ursin G, et al. Dietary patterns and breast cancer risk in the California Teachers Study cohort. Am J Clin Nutr. 2013;98(6):1524-32.
21. Pacheco LS, Lacey JV, Jr., Martinez ME, Lemus H, Araneta MRG, Sears DD, et al. Sugar-sweetened beverage intake and cardiovascular disease risk in the California Teachers Study. J Am Heart Assoc. 2020;9(10):e014883.
22. Reynolds P, Hurley SE, Hoggatt K, Anton-Culver H, Bernstein L, Deapen D, et al. Correlates of active and passive smoking in the California Teachers Study cohort. J Womens Health (Larchmt). 2004;13(7):778-90.
23. Story M, Mays RW, Bishop DB, Perry CL, Taylor G, Smyth M, et al. 5-a-day power plus: process evaluation of a multicomponent elementary school program to increase fruit and vegetable consumption. Health Educ Behav. 2000;27(2):187-200.
24. Chrisman M, Patel S, Alonzo R. Barriers to and facilitators of using MyPlate nutritional guidelines in K-12 teachers and principals. Health Educ J. 2019;79(2):152-65.
25. Frerichs L, Brittin J, Intolubbe-Chmil L, Trowbridge M, Sorensen D, Huang TT. The role of school design in shaping healthy eating-related attitudes, practices, and behaviors among school staff. J Sch Health. 2016;86(1):11-22.
26. LeCheminant JD, Merrill RM, Masterson T. Health behaviors and work-related outcomes among school employees. Am J Health Behav. 2015;39(3):345-51.
27. LeCheminant J, Merrill RM, Masterson TD. Changes in behaviors and outcomes among school-based employees in a wellness program. Health Promot Pract. 2017;18(6):895-901.
28. Merrill RM, Sloan A. Effectiveness of a health promotion program among employees in a Western United States school district. J Occup Environ Med. 2014;56:639–44.
29. Kubik MY LL, Hannan PJ, Story M, Perry CL. Food-related beliefs, eating behavior, and classroom food practices of middle school teachers. J Sch Health. 2002;72(8):339-45.
30. Rossiter M, Glanville T, Taylor J, Blum I. School food practices of prospective teachers. J Sch Health. 2007;77(10):694-700.
31. Hartline-Grafton HL, Rose D, Johnson CC, Rice JC, Webber LS. The influence of weekday eating patterns on energy intake and BMI among female elementary school personnel. Obesity (Silver Spring). 2010;18(4):736-42.
32. Hartline-Grafton HL, Rose D, Johnson CC, Rice JC, Webber LS. Are school employees role models of healthful eating? Dietary intake results from the ACTION worksite wellness trial. J Am Diet Assoc. 2009;109(9):1548-56.
33. Hartline-Grafton HL, Rose D, Johnson CC, Rice JC, Webber LS. Energy density of foods, but not beverages, is positively associated with body mass index in adult women. Eur J Clin Nutr. 2009;63(12):1411-8.
34. Sirajuddin S M, Salam A. The effect of giving rice bran milk on blood glucose levels and body weight in hyperglycemic primary school teachers in Makassar City. Int J Pharm Res. 2021;13(1).
35. Parker EA, Feinberg TM, Lane HG, Deitch R, Zemanick A, Saksvig BI, et al. Diet quality of elementary and middle school teachers is associated with healthier nutrition-related classroom practices. Prev Med Rep. 2020;18:101087.
36. Schultz NS, Chui KKH, Economos CD, Lichtenstein AH, Volpe SL, Sacheck JM. Impact of physical activity, diet quality and stress on cardiometabolic health in school employees. Prev Med Rep. 2020;20:101243.
37. Delfino LD, Tebar WR, Gil FC, De Souza JM, Romanzini M, Fernandes RA, et al. Association of sedentary behaviour patterns with dietary and lifestyle habits among public school teachers: a cross-sectional study. BMJ Open. 2020;10(1):e034322.
38. Berger-Jenkins E, Rausch J, Okah E, Tsao D, Nieto A, Lyda E, et al. Evaluation of a coordinated school-based obesity prevention program in a hispanic community: choosing healthy and active lifestyles for kids/healthy schools healthy families. Am J Health Educ. 2014;45(5):261-70.
39. Lee BR, Ko YM, Cho MH, Yoon YR, Kye SH, Park YK. Effects of 12-week vegetarian diet on the nutritional status, stress status and bowel habits in middle school students and teachers. Clin Nutr Res. 2016;5(2):102-11.
40. Al-Refaee F, Al-Dhafiri S, Al-Qattan S, Al-Mutairi A, Jaber S, Nassar M. Nutritional knowledge, attitude and practice of high school girls living in Kuwait: a pilot study. Kuwait Med J. 2013;45:118-22.
41. Snelling A, Belson SI, Young JL. School health reform: investigating the role of teachers. J Child Nutr Manag. 2012;36.
42. Kinsler J, Slusser W, Erausquin JT, Thai C, Prelip M. Nutrition knowledge and self-efficacy among classroom teachers from a large urban school district in Los Angeles County. Calif J Health Promot. 2012;10:118-25.
43. Kaschalk-Woods E, Fly AD, Foland EB, Dickinson SL, Chen X. Nutrition curriculum training and implementation improves teachers' self-efficacy, knowledge, and outcome expectations. J Nutr Educ Behav. 2021;53(2):142-50.
44. Dalais L, Abrahams Z, Steyn N, Villiers A, Fourie J, Hill J, et al. The association between nutrition and physical activity knowledge and weight status of primary school educators. S Afr J Educ. 2014;34.
45. Findholt NE, Izumi BT, Shannon J, Nguyen T. Food-related practices and beliefs of rural US elementary and middle school teachers. Rural Remote Health. 2016;16(2):3821.
46. Jones AM, Zidenberg-Cherr S. Exploring nutrition education resources and barriers, and nutrition knowledge in teachers in California. J Nutr Educ Behav. 2015;47(2):162-9.
47. Antwi J, Ohemeng A, Boateng L, Quaidoo E, Bannerman B. Primary school-based nutrition education intervention on nutrition knowledge, attitude and practices among school-age children in Ghana. Glob Health Promot. 2020;27(4):114-22.
48. Motamedrezaei O, Moodi M, Miri MR, Khodadadi M. The effect of nutrition and food hygiene education on the knowledge of female elementary school teachers in city of ferdows. J Educ Health Promot. 2013;2:10.
49. Katsagoni CN, Apostolou A, Georgoulis M, Psarra G, Bathrellou E, Filippou C, et al. Schoolteachers’ nutrition knowledge, beliefs, and attitudes before and after an e-learning program. J Nutr Educ Behav. 2019;51(9):1088-98.
50. Husain W, Ashkanani F, Al Dwairji MA. Nutrition knowledge among college of basic education students in Kuwait: a cross-sectional study. J Nutr Metab. 2021;2021:5560714.
51. Hamilton L, Goodman L, Roberts L, Dial LA, Pratt M, Musher-Eizenman D. Teacher experience, personal health, and dieting status is associated with classroom health-related practices and modeling. J Sch Health. 2021;91(2):155-63.
52. O’Dea J. Evaluation of nutrition and physical activity knowledge, attitudes, self efficacy and behaviors in teachers and children after implementation of the “Healthy Active Kids” online program in Australian elementary schools. Health. 2016;08:293-303.
53. Rafiroiu AC EA. Nutrition knowledge, attitudes, and practices among nutrition educators in the south. Am J Health Stud 2005;20(1).
54. Gaglianone C, Taddei J, Colugnati F, Magalhães C, Davanço G, de Macedo L, et al. Nutrition education in public elementary schools of São Paulo, Brazil: the reducing risks of illness and death in adulthood project. Rev Nutr. 2006;19.
55. Shi-Chang X, Xin-Wei Z, Shui-Yang X, Shu-Ming T, Sen-Hai Y, Aldinger C, et al. Creating health-promoting schools in China with a focus on nutrition. Health Promotion International. 2004;19(4):409-18.
56. Wilna O-T, Egal A. Impact of nutrition education on nutrition knowledge of public school educators in South Africa: a pilot study. Health SA Gesondheid. 2012;17.
57. Linnell JD, Smith MH, Briggs M, Brian KM, Scherr RE, Dharmar M, et al. Evaluating the relationships among teacher characteristics, implementation factors, and student outcomes of children participating in an experiential school-based nutrition program. Pedagogy Health Promot. 2016;2(4):256-65.
58. Hasan N, Hadju V, Jafar N, Thaha R. A relationship between knowledge, attitude, and practice about balanced nutrition guidelines and metabolic syndrome among central obese teachers in Makassar. Indian J Public Health Res Dev. 2019;10:579.
59. Falkenbach D, D'Avila H, Mello E. Knowledge, attitudes and practices of primary school teachers on nutrition and food. International Journal of Nutrology. 2018;11:021-9.
60. Hyska J, Burazeri G, Menza V, Dupouy E. Assessing nutritional status and nutrition-related knowledge, attitudes and practices of Albanian schoolchildren to support school food and nutrition policies and programmes. Food Policy. 2020;96:101888.
61. Shah P, Misra A, Gupta N, Hazra D, Gupta R, Seth P, et al. Improvement in nutrition-related knowledge and behaviour of urban Asian Indian school children: findings from the Medical education for children/Adolescents for Realistic prevention of obesity and diabetes and for healthy aGeing (MARG) intervention study. Br J Nutr. 2010;104:427-36.
62. Lai Yeung Wai-ling T. Combating deceptive advertisements and labelling on food products – an exploratory study on the perceptions of teachers. Int J Consum Stud. 2004;28(2):117-26.
63. Molloy CJ, Gandy J, Cunningham C, Slattery G. An exploration of factors that influence the regular consumption of water by Irish primary school children. J Hum Nutr Diet. 2008;21(5):512-5.
64. Kupolati MD, MacIntyre UE, Gericke GJ, Becker P. A contextual Nutrition education program improves nutrition knowledge and attitudes of South African teachers and learners. Front Public Health. 2019;7.
65. Okeyo AP, Seekoe E, de Villiers A, Faber M, Nel JH, Steyn NP. The food and nutrition environment at secondary schools in the Eastern Cape, South Africa as reported by learners. Int J Environ Res Public Health. 2020;17(11).
66. Hemati M, Toori M, Shams M, Behroozpour A. Effect of an educational intervention on nutrition literacy in teachers: a short communication. Malays J Nutr. 2020;26:495-500.
67. Barwood D, Smith S, Miller M, Boston J, Masek M, Devine A. Transformational game trial in nutrition education. Aust J Teach Educ. 2020;45:18-29.
68. Jafar N, Hasan N, Hadju V, Thaha RM, Arundhana AI. Improved knowledge, attitudes, and practices of balanced nutrition after educational intervention based on the self-determination theory: an Intervention study in senior school teachers in Makassar City. Open Access Maced J Med Sci. 2020;8(E):228-35.
69. Both J, Borgatto A, Sonoo C, Lemos C, Ciampolini V, Vieira J. Multiple jobholding associated with the wellbeing of physical education teachers in Southern Brazil. Educación Fisica y Deporte. 2016;35:1-14.
70. Both J, Borgatto A, Lemos C, Ciampolini V, Vieira J. Physical education teachers’ wellbeing and its relation with gender. Motricidade. 2017;13:23-32.
71. Coccia CC, Tamargo J, Macchi AK. Effects of nutrition knowledge, personal health and self-efficacy on food-related teaching practices of elementary school pre-service teachers. Health Educ J. 2020;79(8):974-86.
72. Metos JM, Sarnoff K, Jordan KC. Teachers' perceived and desired roles in nutrition education. J Sch Health. 2019;89(1):68-76.
73. Ates H. Elementary school teachers’ behavioral intentions for healthy nutrition. Health Educ. 2019;119(2):133-49.
74. Gebretatyos H, Amanuel S, Ghirmai L, Gebreyohannes G, Tesfamariam EH. Effect of health education on healthy nutrition and physical activity among female teachers aged 40-60 years in Asmara, Eritrea: a quasiexperimental study. J Nutr Metab. 2020;2020:5721053.
75. Lemon SC, Liu Q, Magner R, Schneider KL, Pbert L. Development and validation of worksite weight-related social norms surveys. Am J Health Behav. 2013;37(1):122-9.
76. O'Dea JA, Abraham S. Knowledge, beliefs, attitudes, and behaviors related to weight control, eating disorders, and body image in Australian trainee home economics and physical education teachers. J Nutr Educ. 2001;33(6):332-40.
77. Vio F, Yañez M, González CG, Fretes G, Salinas J. Teachers' self-perception of their dietary behavior and needs to teach healthy eating habits in the school. J Health Psychol. 2018;23(8):1019-27.
78. Wilkinson C, Prusak K, Johnson T. Self-regulation of physical education teacher education students’ attitudes towards exercise and diet. ICHPER SD Journal of Research. 2013;8:49-54.
79. Thaha RM, Hasan N, Hadju V, Jafar N, Muhiddin S, Maria IL. Measuring self-regulation after nutrition education modules with Self Determination Theory (SDT) intervention among teachers with or at risk metabolic syndrome. Gac Sanit. 2021;35 Suppl 1:S83-s6.
80. Woynarowska-Soldan M. Project on school staff health promotion in Poland: the first experiences. Health Education. 2015;115(3/4):405-19.
81. Laurie SM, Faber M, Maduna MM. Assessment of food gardens as nutrition tool in primary schools in South Africa. South Afr J Clin Nutr. 2017;30(4):80-6.
82. Thompson BM, Ribera KP, Wingenbach GJ, Vestal TA. The relationship between attitudes, knowledge, and demographic variables of high school teachers regarding food irradiation. J Food Sci Educ. 2007;6(2):24-9.
83. Elorinne A-L, Eronen L, Pollari M, Hokkanen J, Reijonen H, Murphy J. Investigating home economics teachers' food waste practices and attitudes. J Teach Educ Sustain. 2020;22:6-20.
84. Prescott M, Lohse B, Balgopal M, Smith S, Addington R, Cunningham-Sabo L. Teacher well-being attributes are positively associated with teacher perceptions of Fuel for Fun tasting lessons. Top Clin Nutr. 2018;33:272-80.
